# Comparative Validation and Performance of the Barcelona- and ERSPC-MRI Predictive Model in an Ibero-American Population of Men Suspected of Having Prostate Cancer

**DOI:** 10.3390/cancers18162541

**Published:** 2026-08-07

**Authors:** Nahuel Paesano, Violeta Catalá, Juan Camean, Tomás Eduardo Olmedo, Joaquín Ignacio Gurovich, Maximiliano Ringa, Berta Miró, Lucas Regis, Olga Mendez, Enrique Trilla, Juan Morote

**Affiliations:** 1 Uro-Radiology Unit, Clínica Creu Blanca, 08034 Barcelona, Spain; nahuel.paesano@rocclinic.com (N.P.); viletacatala@uroima.com (V.C.); 2 Department of Surgical Oncology, Instituto Alexander Fleming, Buenos Aires 1426, Argentina; jcamean@alanderfleming.org; 3 Department of Urology, Hospital Clínico Universidad de Chile, Independencia, Santiago 8380456, Chile; tolmedo@hcucgh.cl (T.E.O.); joaquin.gurovich@ug.uchile.cl (J.I.G.); 4 Department of Urology, Hospital Alemán, Buenos Aires 1424, Argentina; mringa@hospitalaleman.com; 5 Bioinformatics and Statistical Unit, Vall d’Hebron Research Institute, 08035 Barcelona, Spain; bertamiro@vhio.net; 6 Research Group in Urology, Vall d’Hebron Research Institute, 08035 Barcelona, Spain; lucas.regis@vallhebron.cat (L.R.); enrique.trilla@vallhebron.cat (E.T.); 7 Department of Urology, Hospital Universitario Vall d’Hebron, 08035 Barcelona, Spain; 8 Department of Surgery, Universitat Autònoma de Barcelona, 08193 Bellaterra, Spain

**Keywords:** clinically significant prostate cancer, predictive model, magnetic resonance imaging

## Abstract

**Background**: External validation of predictive models is needed in new populations after their implementation. **Objective**: We aimed to validate and compare the performance of the BCN-MRI PM and ERSPC-MRI PM in an Ibero-American population. **Methods**: We conducted a retrospective analysis of 540 men with suspected PCa who underwent MRI and prostate biopsy in 2025. Individual csPCa risk estimates were calculated using the online calculators for the BCN-MRI PM and ERSPC-MRI PM. The reference standard outcome was the rate of avoided biopsies. Calibration, discrimination of csPCa, net benefit, and clinical utility were analyzed with R software. **Results**: All participants underwent MRI and prostate biopsy. The median age was 66 years (95% CI 61–71). The overall csPCa detection rate was 55.7%. The BCN-MRI PM demonstrated good calibration, with close agreement between predicted and observed csPCa probabilities, whereas the ERSPC-MRI PM underestimated risk in the intermediate-probability range. Discrimination for csPCa was significantly better with the BCN-MRI PM (AUC, 0.807; 95% CI 0.771–0.843) than with the ERSPC-MRI PM (AUC, 0.764; 95% CI 0.724–0.803; *p* < 0.001). Decision curve analysis showed a greater net benefit for the BCN-MRI PM than for the ERSPC-MRI PM and the strategy of biopsying all men. Clinical utility curves demonstrated a more favorable balance between avoided biopsies and missed csPCa cases across threshold probabilities. At a sensitivity of 95%, the BCN-MRI PM achieved a specificity of 38.9% (95% CI, 32.5–45.1%) compared with 30.1% (95% CI, 24.6–35.7%) for the ERSPC-MRI PM, avoiding 21.5% and 16.3% of biopsies, respectively (*p* = 0.043). The performance of both models was also evaluated according to PI-RADS category and participating center. **Conclusions**: In this Ibero-American cohort, the BCN-MRI PM demonstrated superior calibration and overall clinical performance compared with the ERSPC-MRI PM.

## Simple Summary

Early detection of clinically significant prostate cancer (csPCa) reduces PCa-specific mortality. Predictive models (PMs) may improve the efficacy of csPCa detection by facilitating the appropriate selection of candidates for prostate biopsy. The Barcelona (BCN)-magnetic resonance imaging (MRI) PM (BCN-MRI PM) and the European Randomized Screening for Prostate Cancer (ERSPC)-MRI PM (ERSPC-MRI PM) are two widely used predictive models with freely available online risk calculators. In this study, we validated and compared the clinical performance of the BCN-MRI PM and the ERSPC-MRI PM in an Ibero-American population.

## 1. Introduction

Prostate cancer (PCa) is the second most frequently diagnosed cancer among men worldwide and the third leading cause of cancer-related mortality [1]. Despite its high incidence and substantial health burden, early detection of clinically significant prostate cancer (csPCa) remains a cornerstone of effective disease management [2,3,4]. Timely identification of csPCa enables the initiation of appropriate treatment strategies, which has been shown to reduce prostate cancer-specific mortality and improve patient outcomes [5].

The diagnostic work-up for suspected PCa is typically initiated by an elevated serum prostate-specific antigen (PSA) level, usually >3.0 ng/mL. Magnetic resonance imaging (MRI) is then recommended to estimate the likelihood of csPCa using the prostate imaging reporting and data system (PI-RADS). The risk of csPCa is categorized into five levels: very low (1), low (2), intermediate (3), high (4), and very high (5). Combined targeted and systematic biopsies are generally recommended for men with PI-RADS scores ≥3, whereas systematic biopsy alone may be considered in men with PI-RADS scores <3 who remain at high risk of csPCa because of elevated PSA density, suspicious digital rectal examination (DRE) findings, or persistently rising PSA levels [6,7,8]. Despite this contemporary diagnostic pathway, unnecessary biopsies and overdetection of insignificant PCa (iPCa) remain major challenges [9]. To address these limitations, the European Association of Urology (EAU) recommends risk-adapted diagnostic pathways based on validated predictive models. These approaches are expected to reduce unnecessary MRI examinations and prostate biopsies while improving csPCa detection and minimizing missed csPCa cases [10,11,12,13,14,15,16]. Several MRI-based predictive models have been developed to improve risk stratification and optimize biopsy selection [17]. However, only a few provide individualized risk estimates through freely accessible and clinically applicable risk calculators [18,19]. Moreover, external validation is essential before implementing predictive models in populations other than those in which they were developed and whenever diagnostic pathways evolve over time [20,21].

The European Randomized Study of Screening for Prostate Cancer MRI-based predictive model (ERSPC-MRI PM), also known as the SWOP or Rotterdam MRI model, was developed in 2019 from the previous ERSPC risk calculators 3/4 by incorporating PI-RADS version 1 findings (https://www.prostatecancer-riskcalculator.com) [22]. The model has undergone extensive external validation across diverse populations, but never in the Ibero-American population, frequently requiring local recalibration to maintain optimal performance [23,24,25,26,27,28,29,30]. Following its validation and recalibration in a contemporary Norwegian cohort, the online calculator was updated to reflect a csPCa prevalence of approximately 20% [27]. Consequently, the developers recommend local recalibration whenever cancer detection rates differ substantially from those of the original development cohort.

The Barcelona-MRI predictive model (BCN-MRI PM or BCN-PM 2) was developed in 2022 and incorporated into the Barcelona risk calculator (http://bcnrc.com), together with the BCN-PM 1 developed to be used prior to MRI [31,32]. Unlike the ERSPC-MRI PM, the BCN-MRI PM was developed using PI-RADS version 2.0 and incorporates age, PSA level, and MRI-derived prostate volume without range limitation, and previous negative biopsy, family history of PCa, DRE findings, and PI-RADS score. Since its development, the BCN-MRI PM has been externally validated in several contemporary clinical settings, including men receiving 5-alpha reductase inhibitors, cohorts evaluated using PI-RADS version 2.1, transperineal biopsy pathways, saturation-targeted biopsy protocols, and both European and Ibero-American populations [33,34,35,36,37].

Because individualized csPCa risk estimates generated by the ERSPC-MRI PM were available in three participating centers involved in the external validation of BCN-PM 1 and 2 and the BCN-risk-stratified pathway in an Ibero-American population, we aimed to compare the calibration and clinical performance of both predictive models. The secondary objectives were: (i) to describe the baseline characteristics of the overall study population and assess heterogeneity among participating centers; (ii) to compare model calibration, discrimination, and net benefit for csPCa detection; (iii) to compare the overall clinical effectiveness of both models, particularly at high sensitivity thresholds for csPCa detection; and (iv) to compare the clinical utility of both predictive models according the PI-RADS scores and participant centers.

## 2. Materials and Methods

### 2.1. Design, Setting, and Participants

**Figure 1:**
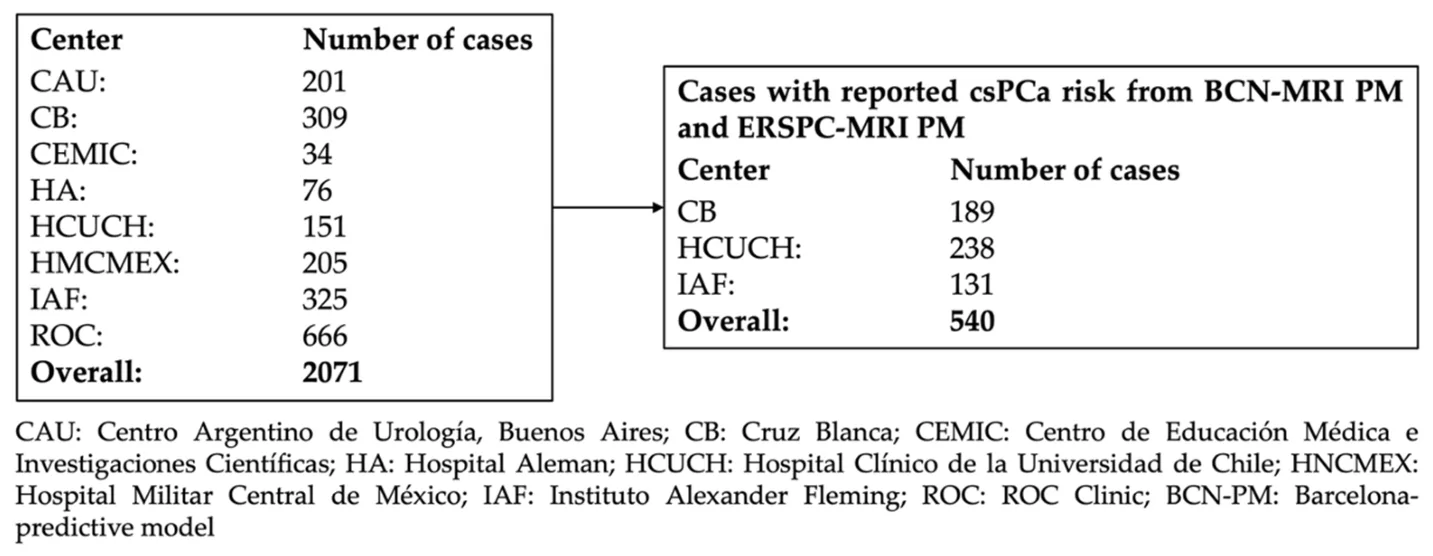
Flowchart of the study cohort used for the assessment of csPCa risk with the BCN-MRI PM and ERSPC-MRI PM.

A retrospective validation and performance comparison of BCN-MRI PM and ERSPC-MRI PM was conducted in an Ibero-American cohort of 540 men with suspected PCa who underwent MRI and prostate biopsy in 2025. This cohort was selected from 2017 men enrolled in a prospective validation study of the BCN-risk-stratified pathway across eight centers in Ibero-America: four in Argentina, two in Spain, one in Chile, and one in México. Instituto Alexander Fleming (IAF) in Buenos Aires (Argentina), Cruz Blanca (CB) in Barcelona (Spain), and Hospital Clínico de la Universidad de Chile (HCUCH) in Santiago (Chile), additionally reported individual csPCa risk estimates derived from the ERSPC-RC for men who underwent MRI, comprising 239, 189, and 113 cases, respectively (Figure 1).

### 2.2. PCa Suspicion and Diagnostic Approach for csPCa

PCa suspicion was identified as a serum PSA level > 3.0 ng/mL and/or a suspicious DRE [8]. All participants underwent multiparametric MRI. A 1.5-Tesla strength field scanner was used at IAF, whereas 3-Tesla were used at CB and HCUCH. A surface phased-array coil was used in all cases. MRI examinations were performed according to the recommendations of the European Society of Urogenital Radiology and included T2-weighted, diffusion-weighted, and dynamic contrast-enhanced sequences [38]. All MRI examinations were interpreted locally by experienced radiologists using PI-RADS v2.1 [39,40]. Cognitive MRI-transrectal ultrasound (TRUS) fusion-guided-biopsy was performed at IAF, whereas CB and HUCH used software-based fusion techniques. All prostate biopsies were performed via the transperineal route. Targeted biopsies of PI-RADS > 3 lesions included 2–4 cores per lesion (up to three lesions) at IAF and HCUCH, whereas mapping at 5 mm intervals was performed at CB. In all centers, targeted biopsies were complemented by a 12-core systematic biopsy. Men with PI-RADS < 3 and a high risk of csPCa, based on elevated PSA density, suspicious DRE findings, or increased PSA level, underwent a 12-core systematic biopsy. All biopsies were performed by experienced local urologists. Biopsy specimens were analyzed in local pathology departments by dedicated uropathologists. CsPCa was defined as an International Society of Urologic Pathology (ISUP) Grade Group of ≥2 [41,42].

### 2.3. Data Variables

Variables included in the BCN- and ERSPC-MRI PM were: age (years), prior negative prostate biopsy, DRE, serum PSA, prostate volume derived from MRI, and PI-RADS. Family history of PCa was also required in the BCB-MRI PM. Additionally, 5-ARI treatment was reported. Participant centers reported an anonymized dataset in Excel to the coordinator center.

### 2.4. Assessment of Individual csPCa Risk Using the BCN-MRI PM and ERSPC-MRI PM

Individual csPCa risks were calculated using the BCN-RC (http://bcnrc.com) and the recalibrated version of the ERSPC-RC (https://www.prostatecancer-riskcalculator.com).

### 2.5. Statistical Analysis

Data were reported according to the Standards of Reporting for MRI-targeted Biopsy Studies (START) guidelines [43], and anonymized datasets were harmonized across participating centers. Continuous variables were summarized as medians and interquartile range (IQR), whereas categorical variables were presented as percentages. Comparisons of continuous variables between two and more than two groups were performed using the Mann–Whitney U test and the Kruskal–Wallis test, respectively. Categorical variables were compared using Pearson’s chi-square test. Correlation between risk estimates was assessed with the Pearson correlation index. Odds ratios (ORs) for clinically significant prostate cancer (csPCa), together with their corresponding 95% confidence intervals (CIs), were calculated when appropriate. Inter-center heterogeneity was evaluated by comparing their baseline characteristics across participating institutions. Model calibration was assessed using calibration plots, calibration-in-the-large, calibration slope, and Brier score metrics. Model discrimination was evaluated using receiver operating characteristic (ROC) curve analysis, with area under the curve (AUC), and compared with DeLong’s test. Net benefit was evaluated with decision curve analysis (DCA). The percentage of avoided biopsies and missed csPCa cases across a range of threshold probabilities was estimated with clinical utility curves (CUCs). Thresholds corresponding to high csPCa sensitivity levels and corresponding specificities and avoided biopsies were compared between models. The clinical performance of both models was also evaluated according to PI-RADS category and participating center using the aforementioned statistical methods. All statistical tests were two-sided, and *p*-values < 0.05 were considered significant. Statistical analyses were performed using R software (version 4.3.2; R Foundation for Statistical Computing, Vienna, Austria).

## 3. Results

### 3.1. Characteristics of the Study Population

The distribution of variables included in the BCN-MRI PM and ERSPC-MRI PM is summarized in Table 1. A family history of PCa, a variable included only in the BCN-MRI PM, was present in 10.9% of participants. A suspicious DRE was observed in 18.9% of participants; however, it was the sole indication for PCa suspicion (PSA level < 3.0 ng/mL) in 15 men (2.8%), among whom seven cases of csPCa (2.3%) were detected. Treatment with 5-alpha reductase inhibitors (5-ARIs) was reported in 24 participants, of whom 11 (45.8%) were diagnosed with csPCa, compared with 290 of 516 participants (56.2%) who were not receiving 5-ARI treatment (*p* = 0.317).

The overall rate of csPCa detection was 55.7%. The distribution of csPCa according to PI-TADS category is presented in Table 2. Notably, 5.2% of participants underwent prostate biopsy with PI-RADS < 3.

### 3.2. Analysis and Correlation of csPCa Probabilities Calculated by the BCN-MRI PM and ERSPC-MRI PM

**Figure 2:**
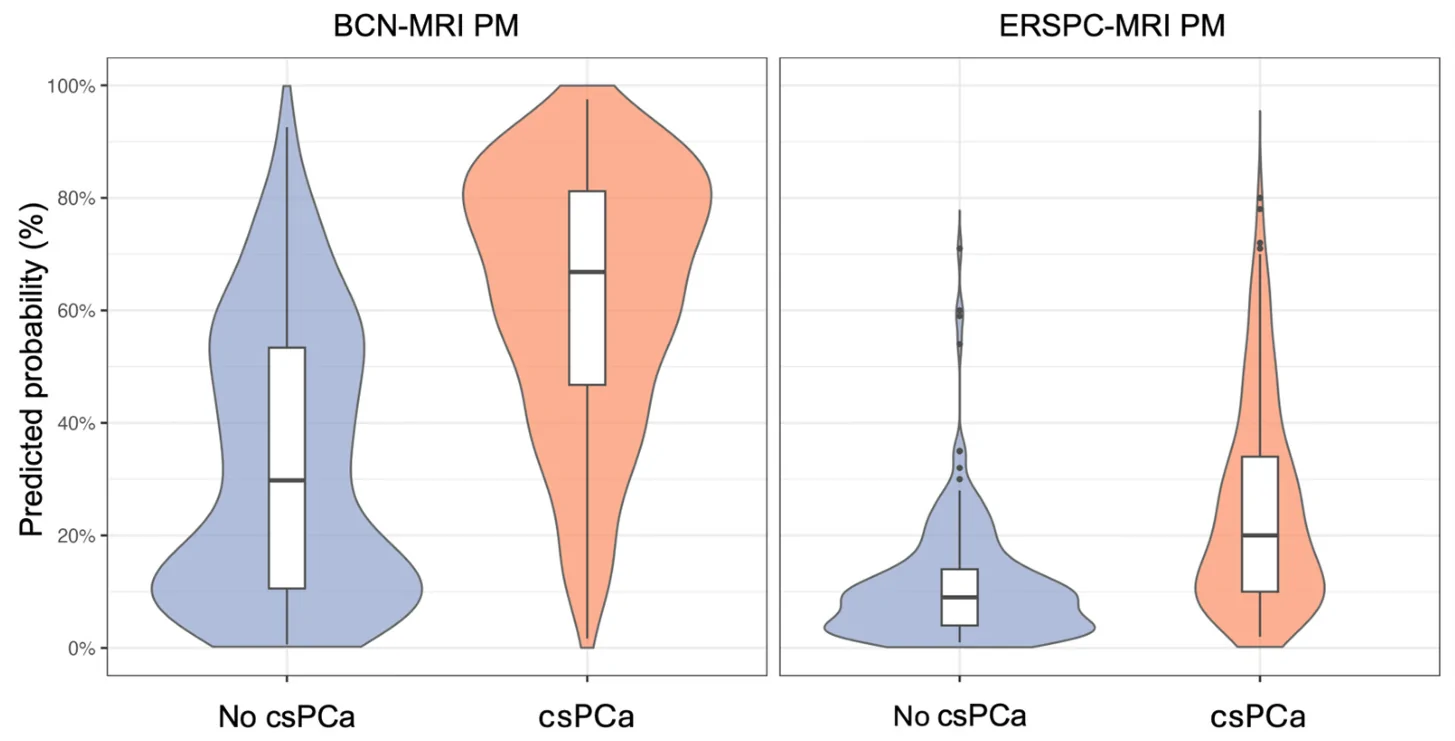
Violin plots illustrating the distribution of predicted csPCa risk in men with and without csPCa detected, assessed with the BCN-MRI PM and ERSPC-MRI PM.

The median probability of csPCa according to the BCN-MRI PM was 29.8% (IQR, 10.5–53.4%) in men without csPCa and 66.8% (IQR, 47.7–81.2%) in those with detected csPCa (*p* < 0.001). The corresponding median probabilities calculated by the ERSPC-MRI PM were 9.1% (IQR, 4.2–14.3%) and 20.2% (IQR, 10.6–34.1%), respectively (*p* < 0.001). Both models yielded significantly higher csPCa predicted risks in men with csPCa than in those without csPCa (*p* < 0.001). However, violin plots demonstrated a broader distribution of predicted risks for the BCN-MRI PM, whereas the ERSPC-MRI PM generated lower risk estimates and showed greater overlap between the two groups (Figure 2).

**Figure 3:**
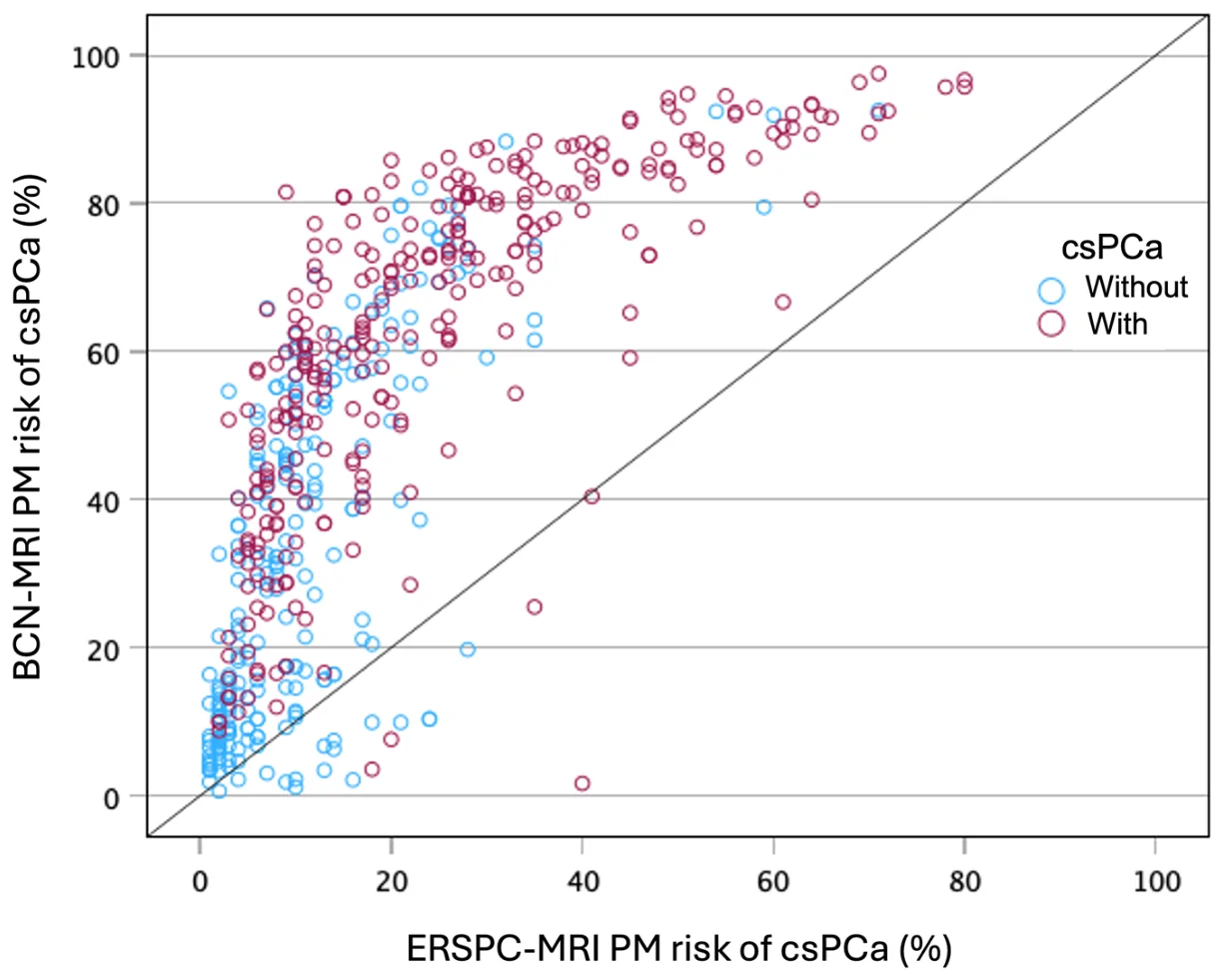
Correlation between csPCa risk estimates generated by the BCN-MRI PM and ERSPC-MRI PM in men with and without csPCa.

The overall correlation between BCN-MRI PM and ERSPC-MRI PM probabilities is presented in Figure 3. The Pearson correlation index was 0.752 (95% CI 0.713–0.797), *p* < 0.001. That means a poor correlation between both csPCa probabilities, even in men with and without csPCa detected. This poor correlation is particularly important within the intermediate-risk range (Figure 3).

### 3.3. Analysis of Heterogeneity Between Participant Centers

Characteristics of each participant center are summarized in Table 3. We note that median age and prostate volume, and percentages of suspicious DRE findings and family history of PCa were similar in the three participant centers. However, median serum PSA and percentages of prior negative biopsy, 5-ARI treatments, and PI-RADS distributions were significantly different. The most important variable was the endpoint csPCa detection rate, which was 64% in CB, 55.5% in IAF, and 41.6% in HCUCH, *p* < 0.001.

### 3.4. Calibration of BCN-MRI PM and ERSPC-MRI PM

**Figure 4:**
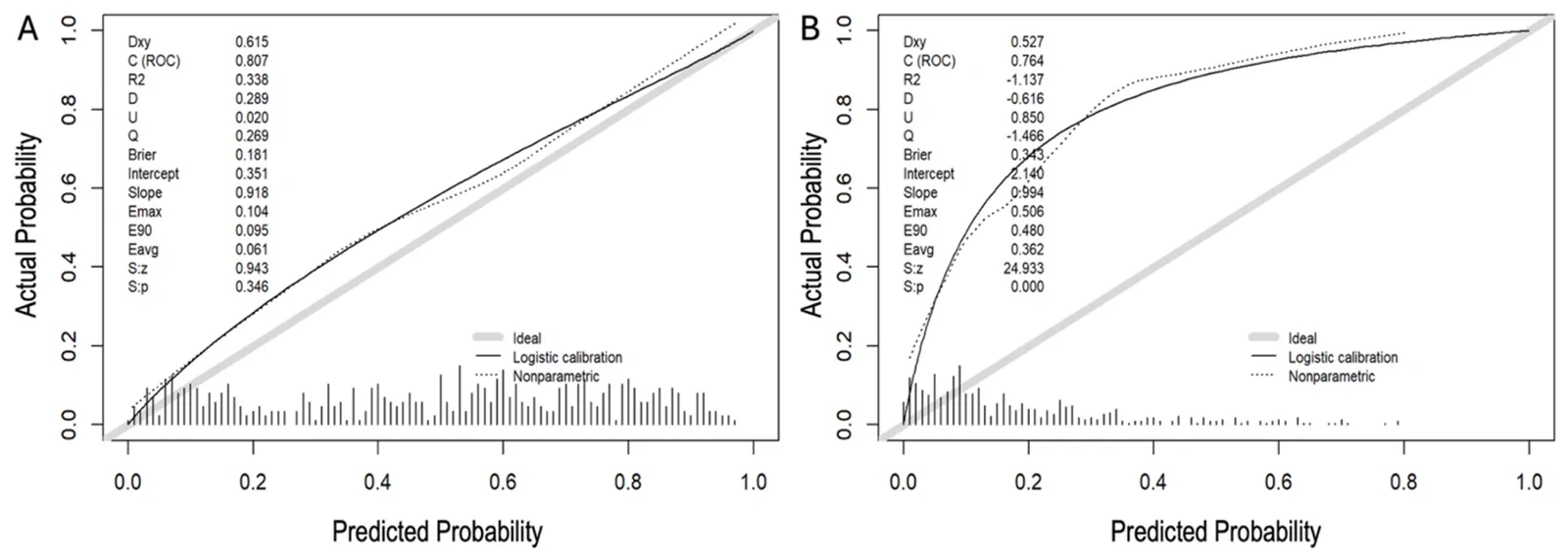
Calibration plots and corresponding calibration metrics for BCN-MRI PM (**A**) and ERSPC-MRI PM (**B**) in predicting csPCa.

Calibration plots and metrics demonstrated good agreement between predicted and observed probabilities of csPCa for the BCN-MRI PM, whereas the ERSPC-MRI PM was substantially miscalibrated, particularly underestimating risk in the intermediate-probability range (Figure 4). For the BCN-MRI PM, the calibration-in-the-large was 0.351 (95% CI, 0.122–0.241), and the calibration slope was 0.918 (95% CI, 0.725–1.121). In contrast, the ERSPC-MRI PM showed a CITL of 2.140 (95% CI, 1.920–3.430) and a calibration slope of 0.994 (95% CI, 0.704–1.321).

### 3.5. Discrimination of csPCa from the BCN-MRI PM and ERSPC-MRI PM

**Figure 5:**
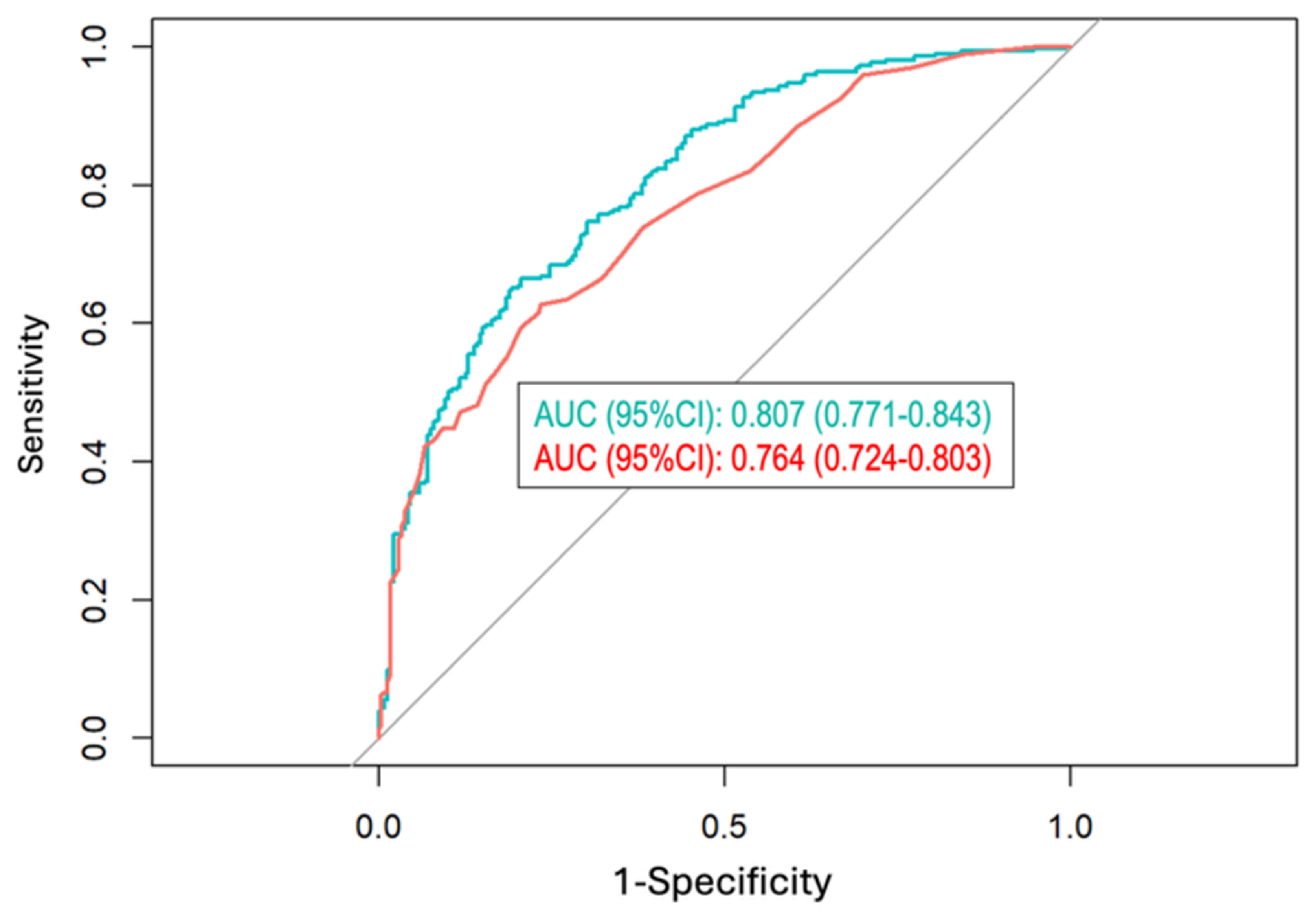
Receiver operating characteristic (ROC) curves showing discrimination of csPCa of BCN-MRI PM and ERSPC-MRI PM.

Discrimination for csPCa was significantly higher for the BCN-MRI PM (AUC of 0.807, 95% CI 0.771–0.843) than for the ERSPC-MRI PM (AUC 0.764, 95% CI 0.724–0.803; *p* < 0.001) (Figure 5).

### 3.6. Net Benefit of BCN-MRI PM and ERSPC-MRI PM

**Figure 6:**
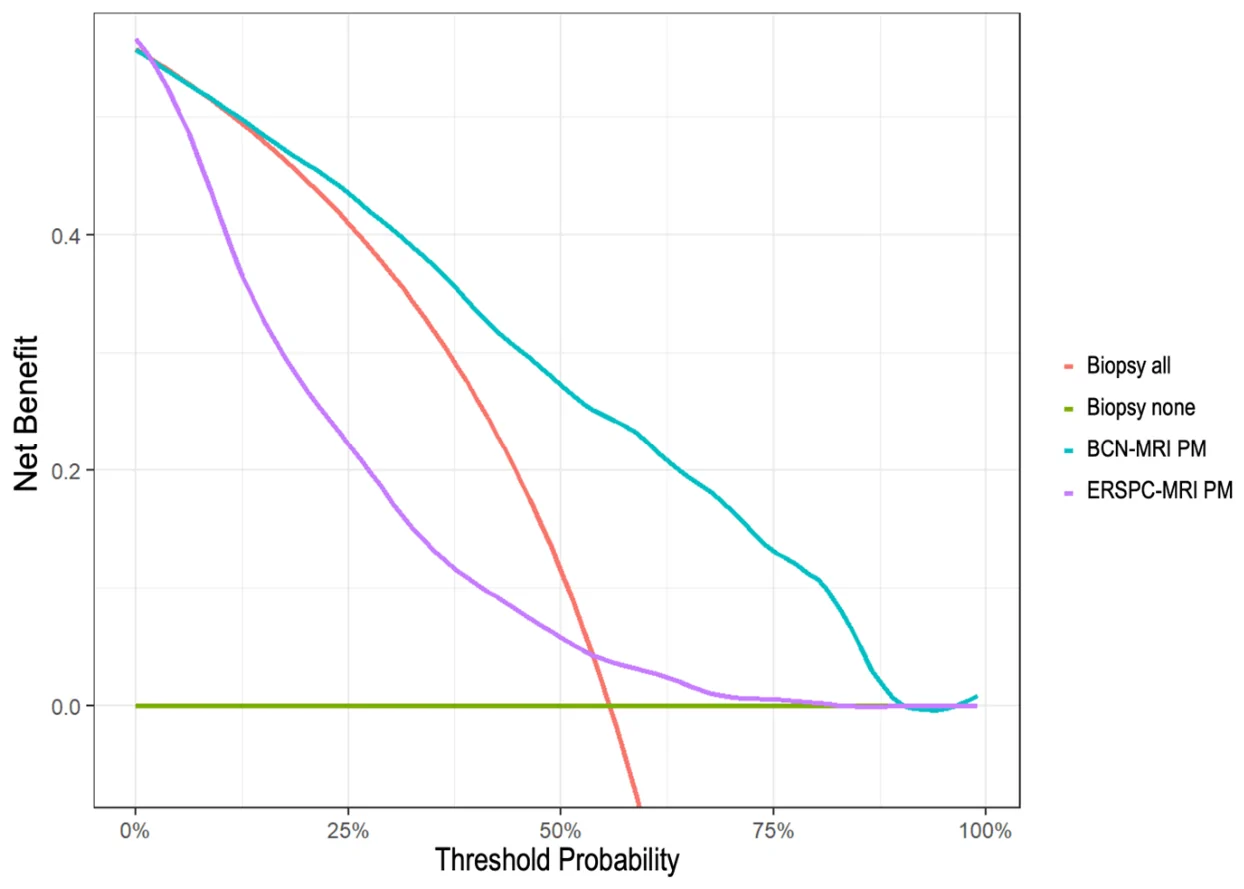
Decision curve analysis (DCA) comparing the net benefit of the BCN-MRI PM and ERSPC-MRI PM for the detection of clinically csPCa across threshold probabilities, relative to the strategies of biopsying all men or no men.

Decision curve analysis (DCAs) demonstrated a greater net benefit for the BCN-MRI PM than for the ERSPC-MRI PM across a wide range of threshold probabilities (Figure 6). The BCN-MRI PM provided a net benefit compared with the strategy of biopsying all men beginning at a 10% threshold probability of csPCa and maintained this benefit across higher threshold probabilities. In contrast, the ERSPC-MRI PM showed a smaller net benefit and only outperformed the biopsy-all strategy at threshold probabilities above 56%.

### 3.7. Clinical Utility of BCN-MRI PM and ERSPC-MRI PM

**Figure 7:**
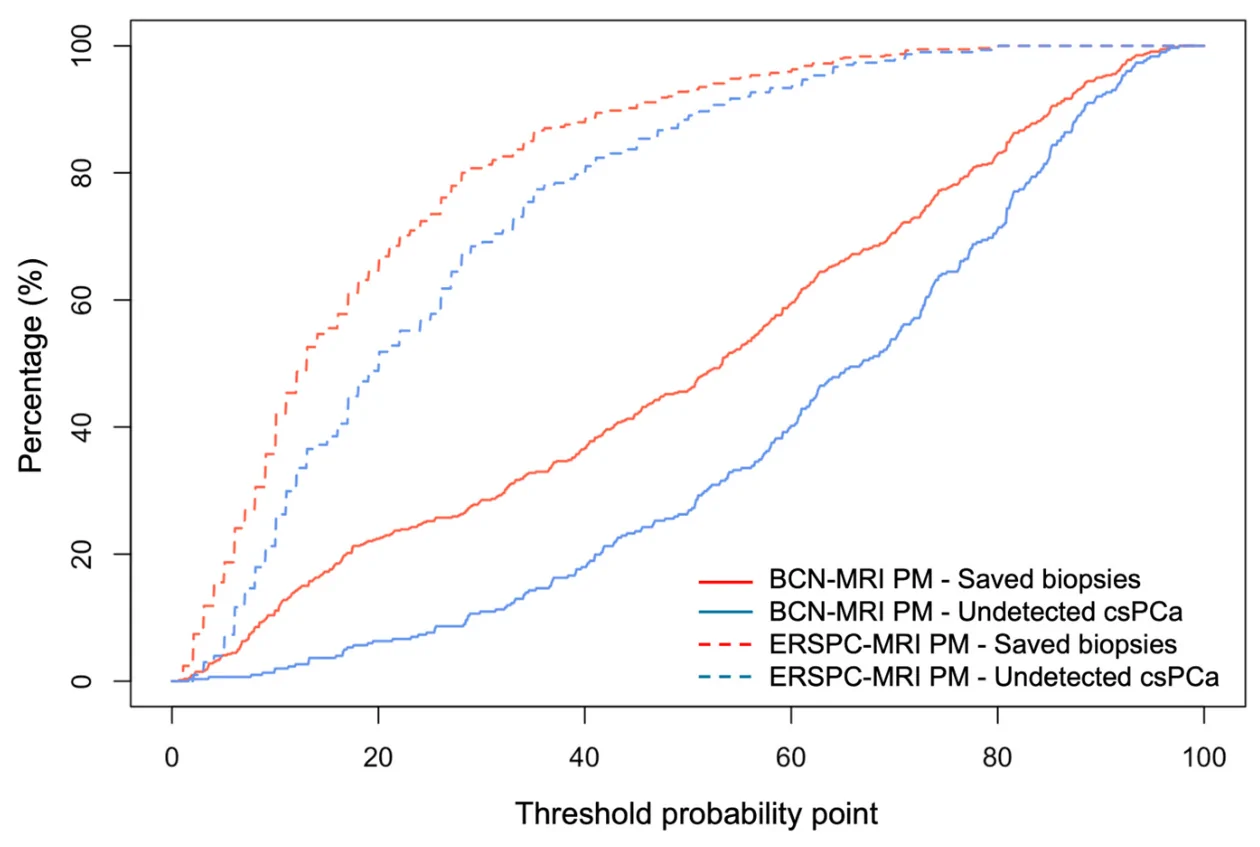
Clinical utility curves (CUCs) showing the proportions of saved biopsies and missed csPCa cases across the range of threshold probability points.

Clinical utility curves (Figure 7) represent the rates of avoided biopsies and missed csPCa cases across the continuous threshold probability points. It is visually observed that the area between the curves of the BCN-MRI PM is greater than that of ERSPC-MRI PM. Supplementary Table S1 presents the rates of avoided biopsies and missed csPCa corresponding to each 5% increase in the threshold probability point.

From the clinical point of view, an important challenge is to miss the less acceptable rate of csPCa detection. Comparison of the efficacy of both predictive models can be assessed by fixing a selected sensitivity to assess the specificity and rates of avoided biopsies. Specificities at high sensitivity thresholds can be clinically relevant because minimizing missed cases of csPCa is critical when selecting an appropriate threshold probability for recommending prostate biopsy. Table 4 compares the specificities of the BCN-MRI PM and ERSPC-MRI PM at sensitivity levels of 100%, 95%, and 90%. The BCN-MRI PM demonstrated higher specificities at the 95% and 90% sensitivity thresholds; however, these differences did not reach statistical significance.

A sensitivity threshold of 95% ensures that no more than 5% of csPCa cases are missed by either predictive model. At this sensitivity level, a 17% risk threshold for the BCN-MRI PM avoided 21.5% of biopsies, whereas a 5% risk threshold for the ERSPC-MRI PM avoided 16.3% of biopsies, both at the cost of missing 5% of csPCa cases (*p* = 0.043).

### 3.8. Performance and Clinical Utility of the BCN-MRI PM and ERSPC-MRI PM According to PI-RADS Categories

Although the overall utility of the BCN-MRI PM and ERSPC-MRI PM has been established in the study cohort, their performance across PI-RADS categories remains essential. Supplementary Figure S1 presents the violin plots of both risk distributions of men with and without csPCa detected at each PI-RADS category. Similar findings to those in the overall cohort distribution are seen at each PI-RADS category, although the risk across them increased. ERSPC-MRI PM risks were lower than BCN-MRI PMs, with high overlap between those in men with and without csPCa.

In PI-RADS 2, due to the low number of cases (28 with two csPCa cases detected), only discrimination analysis could be performed, showing the BCN-MRI PM an AUC of 0.904 (95% 0.741–1.000) compared with 0.694 (95% CI 0.162–1.000), but they were not compared (Supplementary Figure S2). In PI-RADS 3, the AUC of BCN-MRI PM was 0.829 (0.740–0.918) compared to 0.672 (95% CI 0.563–0.780), *p* < 0.001 (Supplementary Figure S3). BCN-MRI PM demonstrated net benefit over biopsy for all men from the 5% probability threshold, while the ERSPC did not show net benefit (Supplementary Figure S4). CUCs demonstrated a higher area between avoided biopsies and missed csPCa cases for the BCN-MRI PM compared with that of ERSPC-MRI PM (Supplementary Figure S5). In PI-RADS 4, the AUC of BCN-MRI PM was 0.696 (0.759–0.635) compared to 0.666 (95% CI 0.729–0.603), *p* = 0.279 (Supplementary Figure S6). BCN-MRI PM demonstrated net benefit over biopsy for all men from the 40% probability threshold, while the ERSPC did not show net benefit (Supplementary Figure S7). CUCs demonstrated a slightly bigger area between avoided biopsies and missed csPCa cases for the BCN-MRI PM compared with that of ERSPC-MRI PM (Supplementary Figure S8). In PI-RADS 5, the AUC of BCN-MRI PM was 0.722 (0.842–0.602) compared to 0.681 (95% CI 0.574–0.799), *p* = 0.278 (Supplementary Figure S9). BCN-MRI PM demonstrated net benefit over biopsy for all men from the 60% probability threshold, while the ERSPC did not show net benefit (Supplementary Figure S10). CUCs demonstrated a slightly larger area between avoided biopsies and missed csPCa cases for the BCN-MRI PM compared with that of ERSPC-MRI PM (Supplementary Figure S11).

The rates of avoided biopsies and missed csPCa cases using the overall 95% sensitivity thresholds are shown below (Table 5).

**Table 5:** Saved biopsies and missed csPCa cases according to PI-RADS categories, using the corresponding 95% sensitivity thresholds for the BCN-MRI PM and ERSPC-MRI PM.

PI-RADS Score	BCN-MRI PM (17% Threshold)		ERSPC-MRI PM (5% Threshold)	
	Saved Biopsies n (%)	Missed csPCa n (%)	Saved Biopsies n (%)	Missed csPCa n (%)
2	17/28 (60.7)	1/2 (50)	11/28 (38.3)	1/2(50)
3	70/98 (71.4)	8/25 (32.0)	62/98(63.3)	11/25(44.0)
4	18/277 (6.5)	4/162 (2.5)	15/277 (5.4)	3/162 (1.9)
5	11/137 (6.0)	2/112 (1.8)	0/137 (0)	0/112 (0)
All	116/540 (21.5)	15/301 (5.0)	88/540 (16.3)	15/301 (5.0)

However, to assess and compare the true clinical utility of BCN-MRI PM and ERSPC-MRI PM, we need to fix the appropriate specific sensitivities for csPCa detection across PI-RADS, which detects the overall 95% sensitivity (Table 6). We began establishing a 100% sensitivity for PI-RADS 5, which was reached only by the BCN-MRI PM, avoiding 1.5% of biopsies. We considered establishing 97.5% sensitivity in PI-RADS 4, avoiding 7.6% of biopsies compared to 4.3% using ERSPC-PM. Thereafter, we considered that 50% sensitivity for previously screened PI-RADS 2 was fine, resulting in 96.4% avoided biopsies for the BCN-MRI PM compared with 89.3% for ERSPC-MRI PM. Finally, we looked for the remaining 60% sensitivity to be applied in PI-RADS 3, which allows for avoiding 80% of biopsies with the BCN-MRI-PM compared to 64.3% with ERSPC-MRI PM.

### 3.9. Clinical Utility of the BCN-MRI PM and ERSPC-MRI PM According to Participant Centers

Because heterogeneity among participant centers has been previously demonstrated, the clinical utility of the overall 95% sensitivity thresholds established for the BCN-MRI PM and ERSPC-MRI PM was evaluated separately for each center (Table 7). We observed that overall 95% sensitivity thresholds did not correspond to the same sensitivity at each participating center. Nevertheless, the difference between avoided biopsies and missed csPCa cases was consistently greater for the BCN-MRI PM compared to the ERSPC-MRI PM across centers.

Additional analyses of discrimination for csPCa (ROCs and AUCs), net benefit (DCAs) and clinical utility (CUCs) for both models according to participating centers are presented in Supplementary Materials. Discrimination for csPCa was significantly higher for the BCN-MRI PM than for the ERSPC-MRI PM at CB, with an AUC of 0.901 (95% CI, 0.855–0.947) versus 0.838 (95% CI, 0.782–0.893), respectively (*p* = 0.005; Supplementary Figure S12). At IAF, the BCN-MRI PM also demonstrated significantly better discrimination than the ERSPC-MRI PM, with AUCs of 0.813 (95% CI, 0.759–0.867) and 0.747 (95% CI, 0.686–0.808), respectively (*p* = 0.002; Supplementary Figure S13). At HCUCH, discrimination was similar between the two models, with an AUC of 0.669 (95% CI, 0.568–0.769) for the BCN-MRI PM and 0.676 (95% CI, 0.577–0.774) for the ERSPC-MRI PM (*p* = 0.769; Supplementary Figure S14). Decision curve analysis demonstrated a greater net benefit for the BCN-MRI PM than for the ERSPC-MRI PM over the strategy of biopsying all men at CB (Supplementary Figure S15), IAF (Supplementary Figure S16), and HCUCH (Supplementary Figure S17). Likewise, CUCs consistently demonstrated greater clinical utility for the BCN-MRI PM than for the ERSPC-MRI PM across all participating centers (Supplementary Figures S18–S20). Analyses of discrimination for csPCa, net benefit, and clinical utility of the BCN-MRI PM and ERSPC-MRI PM according to both participating center and PI-RADS category were not performed due to the insufficient number of cases.

## 4. Discussion

The present external validation demonstrates that the BCN-MRI PM maintained good calibration and superior clinical performance compared with the ERSPC-MRI PM in an Ibero-American cohort of men undergoing opportunistic screening for clinically significant prostate cancer (csPCa). Although both models showed good discrimination, the BCN-MRI PM achieved a higher AUC, greater net benefit across clinically relevant threshold probabilities, and a more favorable balance between avoiding unnecessary biopsies and maintaining csPCa detection. In contrast, the ERSPC-MRI PM systematically underestimated the probability of csPCa, particularly among men at intermediate predicted risk.

Several factors may explain the poorer calibration of the ERSPC-MRI PM. Although the development cohorts of the BCN-MRI PM and ERSPC-MRI PM had very similar csPCa detection rates (36.4% and 35.9%, respectively) [22,32], important differences existed between those cohorts and the present validation population. Most notably, only 5.2% of biopsied men in the present cohort had PI-RADS <3 lesions, compared with 21.2% and 17.7% in the development cohorts of the BCN-MRI PM and ERSPC-MRI PM, respectively [22,32]. This discrepancy reflects current recommendations to restrict biopsy in men with PI-RADS < 3 MRI findings to those with persistent clinical suspicion of csPCa, such as elevated PSA density, abnormal digital rectal examination findings, or persistently increasing PSA levels. Consequently, men with PI-RADS < 3 lesions represented a highly selected subgroup, which may have contributed to the observed model miscalibration.

Differences in imaging interpretation and biopsy strategy may also have influenced model performance. The ERSPC-MRI PM was developed using PI-RADS version 1.0, whereas the BCN-MRI PM was based on PI-RADS version 2.0, which is more closely aligned with the PI-RADS version 2.1 classification used in the present cohort. Furthermore, transrectal biopsy was used in the development cohorts of both models, whereas the transperineal approach predominated in the present study [22,32]. These methodological differences may have affected lesion characterization and csPCa detection, thereby influencing model calibration.

The different calibration performance of both models was further reflected in the distribution of predicted risks. Although both models generated significantly higher predicted risks in men with csPCa than in those without csPCa, the ERSPC-MRI PM consistently assigned lower predicted probabilities, with a narrower distribution and greater overlap between patients with and without csPCa. This pattern is consistent with its tendency to underestimate csPCa risk, particularly at intermediate predicted probabilities, and was further supported by the correlation analysis comparing risk estimates from both models. Similar findings have previously been reported in an independent external validation of the BCN-MRI PM [29]. In addition, the current version of the ERSPC-RC was recalibrated in a population with a csPCa detection rate of approximately 20% [12], substantially lower than the 55.7% observed in the present validation cohort. This difference in disease prevalence may have further contributed to the systematic underestimation of risk by the ERSPC-MRI PM.

The superior calibration of the BCN-MRI PM translated into greater clinical utility. Decision curve analysis demonstrated consistently higher net benefit across clinically relevant threshold probabilities, whereas the ERSPC-MRI PM provided only marginal benefit over a biopsy-all strategy, particularly at higher thresholds. Clinical utility curves also favored the BCN-MRI PM, indicating a more favorable trade-off between reducing unnecessary biopsies and maintaining csPCa detection. Because screening strategies should prioritize high sensitivity, we further compared both models at a fixed sensitivity of 95% for csPCa detection. Although the required thresholds differed substantially (17% for the BCN-MRI PM and 6% for the ERSPC-MRI PM), the BCN-MRI PM avoided significantly more biopsies (21.5% vs. 16.5%). Nevertheless, these thresholds corresponded to the lower end of the predicted risk spectrum, where calibration and predicted risks of both models were more similar [29]

Beyond the overall comparison between BCN- and ERSPC-MRI PM, stratification according to PI-RADS categories provided additional insights into their clinical applicability. Previous studies have demonstrated that combining clinical variables with PI-RADS assessment improves MRI-based risk stratification [17,18,19,20,21,22,23,24,25,26,27,28], although most validation studies have focused primarily on PI-RADS 3 lesions [44,45,46,47,48,49,50,51,52,53,54,55]. Our findings indicate that evaluating predictive models across the entire PI-RADS spectrum provides a more comprehensive assessment of their clinical value. Notably, the BCN-MRI PM maintained 100% sensitivity in men with PI-RADS 5 lesions while avoiding a small proportion of unnecessary biopsies, whereas the ERSPC-MRI PM did not achieve complete sensitivity in this subgroup. Similar trends favoring the BCN-MRI PM were observed in PI-RADS 4, 3, and 2 lesions. Although these differences were not statistically significant, they consistently suggested greater clinical usefulness of the BCN-MRI PM across all MRI suspicion categories. Moreover, adapting decision thresholds according to PI-RADS category increased overall biopsy avoidance while maintaining an overall sensitivity of 95%, supporting the use of PI-RADS-specific risk-adapted biopsy strategy.

Several limitations should be acknowledged. First, only three participating centers had complete information required to calculate both predictive models, which may have introduced selection bias. Second, the low proportion of biopsied men with PI-RADS < 3 lesions limited the precision of subgroup analyses and reflected contemporary biopsy practices rather than an unselected screening population. Third, heterogeneity among participating centers suggests that center-specific validation stratified by PI-RADS category would provide additional information; however, the number of men within individual centers was insufficient to obtain robust estimates. Uncontrolled confounders, such as variability in MRI scanners, differences in reader expertise, and inter-center reporting variability, were also limitations.

Multi-omics analyses will provide a refined characterization of csPCa detected in prostate biopsies with better correlation in the future with the whole prostate true pathology [56,57,58]. Other emerging scoring systems, such as the PRIMARY score based on PSMA hybrid imaging and the miPSMA score, should also be considered in future studies for evaluating men with suspected prostate cancer [59]. Risk prediction models inevitably reflect the characteristics of the populations and clinical practices in which they were developed and need risk calculators to facilitate their clinical use [60]. Consequently, temporal changes in diagnostic pathways and differences between development and validation cohorts may progressively reduce model calibration, highlighting the need for periodic recalibration and adjustment of decision thresholds [20]. Future developments should focus on dynamic prediction models capable of continuous updating through multicenter data integration [61], machine learning methodologies [62,63], and federated learning frameworks [36,64,65], enabling robust and continuously validated risk prediction across diverse clinical settings.

## 5. Conclusions

The BCN-MRI PM demonstrated good calibration in an Ibero-American population with suspected PCa, whereas the ERSPC-MRI PM underestimated the risk of csPCa, particularly at intermediate predicted probabilities. Although both models showed distinct performance patterns, the ERSPC-MRI PM generated lower and narrower predicted probabilities of csPCa, while the BCN-MRI PM achieved superior discrimination, net benefit, and overall clinical utility. This translated into a more favorable balance between avoided biopsies and missed csPCa cases in the overall study population. After applying PI-RADS-specific risk thresholds to maintain appropriate sensitivity, the BCN-MRI PM consistently outperformed the ERSPC-MRI PM across all PI-RADS categories. Nevertheless, its clinical utility varied according to PI-RADS score, being limited in PI-RADS 5, modest in PI-RADS 4, and greatest in PI-RADS 2 and 3. These findings also highlight the need to adjust decision thresholds according to the characteristics of individual participating centers to ensure optimal diagnostic performance.

## Figures and Tables

**Table 1:** Characteristics of men included in this external validation and comparison of performance of the BCN- and ERSPC-MRI PM.

Characteristic	Measurement
Number of men, n	540
Median age, years (IQR)	66 (61–71)
Median serum PSA, ng/mL (IQR)	7.0 (5.0–9.7)
Abnormal DRE, n (%)	102 (18.9)
Prior negative biopsy, n (%)	103 (19.1)
Family history of PCa, n (%)	59 (10.9)
Median prostate volume derived from MRI, mL (IQR)	50 (36–66)
5-ARI treatment, n (%)	24 (4.4)
MRI at 3 Tesla, n (%)	299 (55.4)
Software MRI-TRUS fusion	301 (55.7)
Transperineal route	540 (100)
PI-RADS v2.1 score	
2, n (%)	28 (5.2)
3, n (%)	98 (18.1)
4, n (%)	277 (51.3)
5, n (%)	137 (25.4)
Detection of csPCa, n (%)	301 (55.7)

n = number; IQR = interquartile range; PSA = prostate specific antigen; DRE = digital rectal examination; MRI = magnetic resonance imaging PI-RADS = prostate imaging reporting and data system; csPCa = clinically significant PCa.

**Table 2:** Relationship between PI-RADS category and csPCa detection in prostate biopsies.

Pathology at Prostate Biopsy	PI-RADS Category				All, n (%)
	2	3	4	5	
csPCa, n (%)	2 (7.1)	25 (25.5)	162 (58.5)	112 (81.8)	301 (55.7)
All, n (%)	28 (5.2)	98 (18.1)	277 (51.3)	137 (25.4)	540 (100)

PI-RADS = prostate imaging reporting and data system; PCa = prostate cancer; csPCa = clinically significant PCa.

**Table 3:** Analysis of heterogeneity among the characteristics of participating centers.

Baseline Characteristic	Center			*p* Value
	CB	IAF	HCUCH	
Number of men, n	189	238	113	-
Median age, years (IQR)	66 (61–73)	66 (60–71)	66 (61–70)	0.431
Median serum PSA, ng/mL (IQR)	6.8 (4.1–9.5)	7.6 (4.3–10.0)	6.4 (4.9–8.5)	0.003
Abnormal DRE, n (%)	35(18.5)	48 (20.2)	19 (18.6)	0.745
Prior negative biopsy, n (%)	52 (27.5)	44 (18.5)	7 (6.2)	<0.001
Family history of PCa, n (%)	28 (14.8)	22 (9.2)	8 (8.0)	0.098
Median prostate volume derived from MRI, mL (IQR)	46 (25–63)	52 (30–70)	50 (28–64	0.130
5-ARI treatment, n (%)	0 (0.0)	11 (4.6)	13 (11.5)	<0.001
PI-RADS score				<0.001
2, n (%)	23 (12.2)	1 (0.4)	4 (3.5)	
3, n (%)	40 (21.2)	38 (16.0)	20 (17.7)	
4, n (%)	77 (40.7)	136 (57.1)	64 (56.6)	
5, n (%)	49 (25.9)	63 (26.5)	25 (22.1	
Overall detection of csPCa, n (%)	121 (64.0)	133 (55.9)	47 (41.6)	<0.001

CB = Clínica Cruz Blanca; IAF = Instituto Alexander Fleming; HCUCH = Hospital Clínico de la Universidad de Chile; n = number; IQR = interquartile range; PSA = prostate-specific antigen; DRE = digital rectal examination; MRI = magnetic resonance imaging; PI-RADS = prostate imaging reporting and data system; csPCa = clinically significant PCa.

**Table 4:** Comparison of the specificities of the BCN-MRI PM and ERSPC-MRI PM at sensitivity levels of 100%, 95%, and 90% for the detection of csPCa.

Target Sensitivity (%)	BCN-MRI PM Specificity (95% CI)	ERSPC-MRI PM Specificity (95% CI)	*p* Value
100	0.8 (0.0–2.1)	5.5 (2.8–8.9)	0.479
95	38.9 (32.5–45.1)	30.1 (24.6–35.7)	0.497
90	48.6 (42.3–55.1)	33.1 (27.7–39.2)	0.491

CI = confidence interval.

**Table 6:** Avoided biopsies and missed csPCa cases according to the PI-RADS categories, using specific thresholds corresponding to 100% sensitivity in PI-RADS 5, 97.5% in PI-RADS 4, 60% for PI-RADS 3, and 50% in PI-RADS 2, which resulted in overall 95% sensitivity.

PI-RADS Score	BCN-MRI PM			ERSPC-MRI PM		
	Threshold (%)	Saved Biopsies n (%)	Missed csPCa n (%)	Threshold (%)	Saved Biopsies n (%)	Missed csPCa n (%)
2	21.1	27/28 (96.4)	1/2 (50)	21	25/28 (89.3)	1/2(50)
3	18.3	78/98 (80.0)	10/25 (40)	5	63/98(64.3)	10/25(40.0)
4	16.4	21/277 (7.6)	4/162 (2.5)	6	12/277 (4.3)	4/162 (2.5)
5	97.5	2/137 (1.5)	0/112(0)	80	0/137(0)	0/112 (0)
All	-	128/540 (23.7)	15/301 (5.0)	-	100/540 (18.5)	15/301 (5.0)

PI-RADS = prostate imaging reporting and data system.

**Table 7:** Avoided biopsies and missed csPCa cases according to participant center using the corresponding overall 95% sensitivity thresholds for the BCN-MRI PM and ERSPC-MRI PM.

PI-RADS Score	BCN-MRI PM (17% Threshold)		ERSPC-MRI PM (5% Threshold)	
	Saved Biopsies n (%)	Missed csPCa n (%)	Saved Biopsies n (%)	Missed csPCa n (%)
CB	53/189 (28.0)	7/121 (5.8)	27/189 (14.3)	2/121 (1.7)
IAF	41/238 (17.2)	6/133 (4.5)	35/238(14.7)	10/133 (7.5)
HCUCH	22/113 (19.5)	2/47 (9.1)	26/113 (23.0)	3/47 (6.4)
All	116/540 (21.5)	15/301 (5.0)	88/540 (16.3)	15/301 (5.0)

## Data Availability

The data presented in this study are available on request from the corresponding author.

## Supplementary Materials

The following supporting information can be downloaded at: https://www.mdpi.com/article/10.3390/cancers18162541/s1, Table S1: Percentage of undetected csPCa and saved biopsies according to a 5% increase threshold for the BCN-MRI PM and ERSPC-MRI PM.; Figure S1. Violin plots showing the distribution of BCN- and ERSPC-MRI PM probabilities for csPCa, in men with and without csPCa, according to PI-RADS category; Figure S2: Discrimination for csPCa by the BCN-MRI PM and ERSPC-MRI PM in men suspected of having PCa with PI-RADS 2; Figure S3: Discrimination for csPCa of the BCN-MRI PM and ERSPC-MRI PM in men suspected of having PCa with PI-RADS 3; Figure S4; Net benefit over biopsying all men or none of BCN-MRI PM and ERSPC-MRI PM, in men with suspected PCa and PI-RADS 3; Figure S5: Clinical utility in terms of avoided prostate biopsies and missed csPCa in men with suspected PCa and PI-RADS 3; Figure S6: Discrimination for csPCa by the BCN-MRI PM and ERSPC-MRI PM in men suspected of having PCa with PI-RADS 4; Figure S7: Net benefit over biopsying all men or none of BCN-MRI PM and ERSPC-MRI PM, in men with suspected PCa and PI-RADS 4; Figure S8: Clinical utility in terms of avoided prostate biopsies and missed csPCa in men with suspected PCa and PI-RADS 4; Figure S9. Discrimination for csPCa by the BCN-MRI PM and ERSPC-MRI PM in men suspected of having PCa with PI-RADS 5; Figure S10: Net benefit over biopsying all men or none of BCN-MRI PM and ERSPC-MRI PM in men with suspected PCa and PI-RADS 5; Figure S11: Clinical utility in terms of avoided prostate biopsies and missed csPCa; Figure S12: Discrimination for csPCa by the BCN-MRI PM and ERSPC-MRI PM in men suspected of having PCa at Creu Blanca; Figure S13: Discrimination for csPCa by the BCN-MRI PM and ERSPC-MRI PM in men suspected of having PCa at Instituto Alexander Fleming; Figure S14; Discrimination for csPCa by the BCN-MRI PM and ERSPC-MRI PM in men suspected of having PCa at Hospital Clínico de la Universidad de Chile; Figure S15: Net benefit of the BCN-MRI PM and ERSPC-MRI PM in men suspected of having PCa at Creu Blanca; Figure S16: Net benefit of the BCN-MRI PM and ERSPC-MRI PM in men suspected of having PCa at Instituto Alexander Fleming; Figure S17: Net benefit of the BCN-MRI PM and ERSPC-MRI PM in men suspected of having PCa at Hospital Clínico de la Universidad de Chile; Figure S18: Clinical utility in terms of avoided prostate biopsies and missed csPCa in men from Cruz Blanca; Figure S19: Clinical utility in terms of avoided prostate biopsies and missed csPCa in men from Instituto Alexander Fleming; Figure S20: Clinical utility in terms of avoided prostate biopsies and missed csPCa in men from Hospital Clínico de la Universidad de Chile.

## Abbreviations

The following abbreviations are used in this manuscript:

AUC	Area under the curve
BCN-MRI PM	Barcelona-MRI predictive model
CI	Confidence interval
csPCa	Clinically significant PCa
CUC	Clinical utility curve
DCA	Decision curve analysis
DRE	Digital rectal examination
EAU	European Association of Urology
ERSPC-MRI PM	European Randomized Screening Prostate cancer MRI predictive model
iPCa	Insignificant PCa
IQR	Interquartile range
MRI	Magnetic resonance imaging
PCa	Prostate cancer
PI-RADS	Prostate Imaging Reporting and Data System
PSA	Prostate-specific antigen
ROC	Receiver operating characteristic
TRUS	Transrectal ultrasound
