# Supplementary material for: Comparative Validation and Performance of the Barcelona- and ERSPC-MRI Predictive Model in an Ibero-American Population of Men Suspected of Having Prostate Cancer
Source: Cancers (Basel). 2026 Aug 7;18(16):2541. doi: 10.3390/cancers18162541 (PMC13511617; doi:10.3390/cancers18162541)
Supplement: Supplementary file 1 [file cancers-18-02541-s001.zip › cancers-4459331-supplementary.pdf]

Table S1. Percentage of undetected csPCa and saved biopsies according to a 5% increase threshold for the BCN-MRI PM and ERSPC-MRI PM.

Threshold (%)	BCN-MRI PM		ERSPC-MRI PM	
	Saved Biopsies (%)	Saved Biopsies (%)	Undetected csPCa (%)	Saved Biopsies (%)
0	0	0	0	0
5	0.66	4.07	3.99	15.56
10	1.99	11.11	21.26	35.74
15	3,65	17.22	37.21	54.63
20	6.31	22.41	48.84	64.63
25	7.64	25.18	58.81	72.41
30	10.96	28.51	68.43	80.74
35	14.29	32.78	75.41	85.00
40	17.94	36.67	79.73	87.96
45	23.59	42.04	83.72	90.19
50	26.58	45.74	88.37	92.78
55	33.22	52.22	91.69	94.81
60	40.20	59.44	93.36	95.93
65	48.50	66.11	96.68	97.96
70	53.82	70.41	97.67	98.52
75	64.12	77.41	99.00	99.44
80	71.09	82,96	99.34	99.63
85	82.39	89.44	100	100
90	92.03	95.00	100	100
95	98.34	99.07	100	100
100	100	100	100	100

csPCa = clinically significant PCa

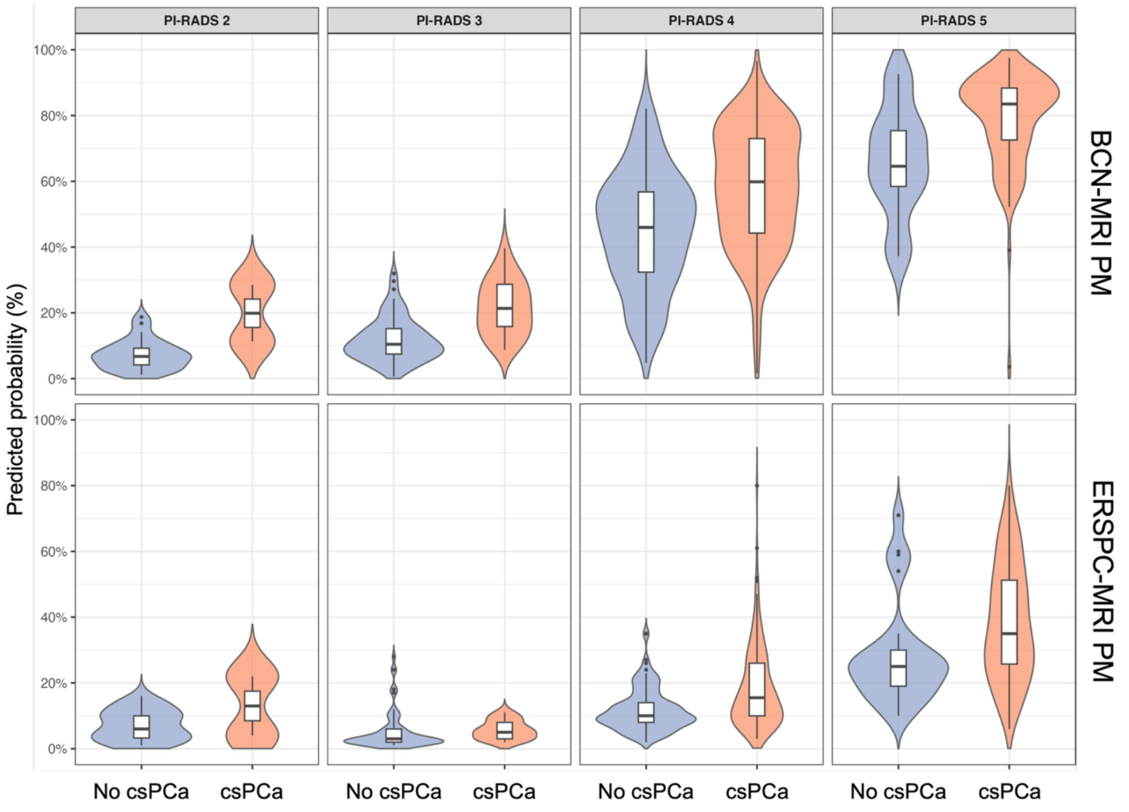

Figure S1. Violin plots showing the distribution of BCN- and ERSPC-MRI PM probabilities for csPCa, in men with and without csPCa, according to PI-RADS category.

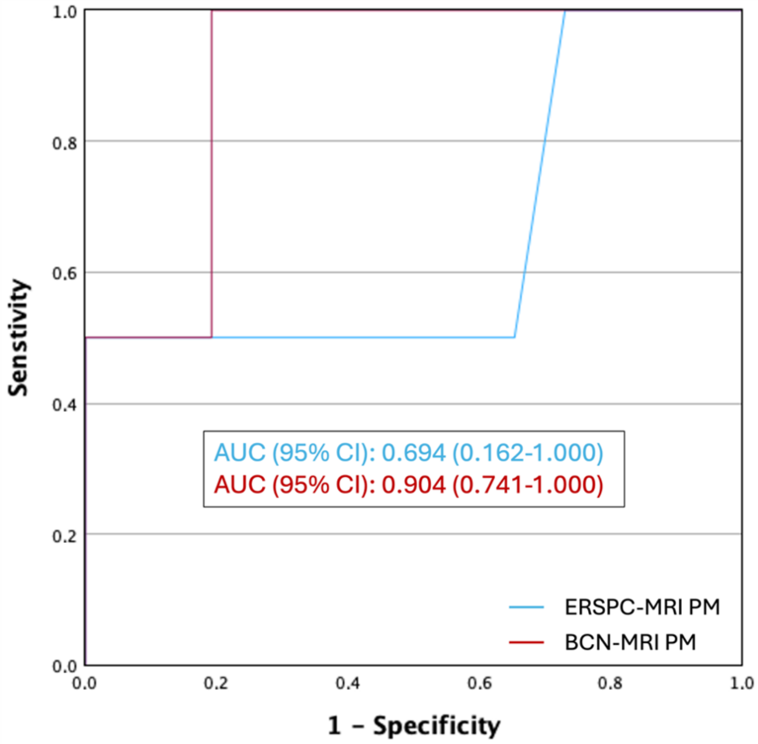

Figure S2. Discrimination for csPCa by the BCN-MRI PM and ERSPC-MRI PM in men suspected of having PCa with PI-RADS 2.

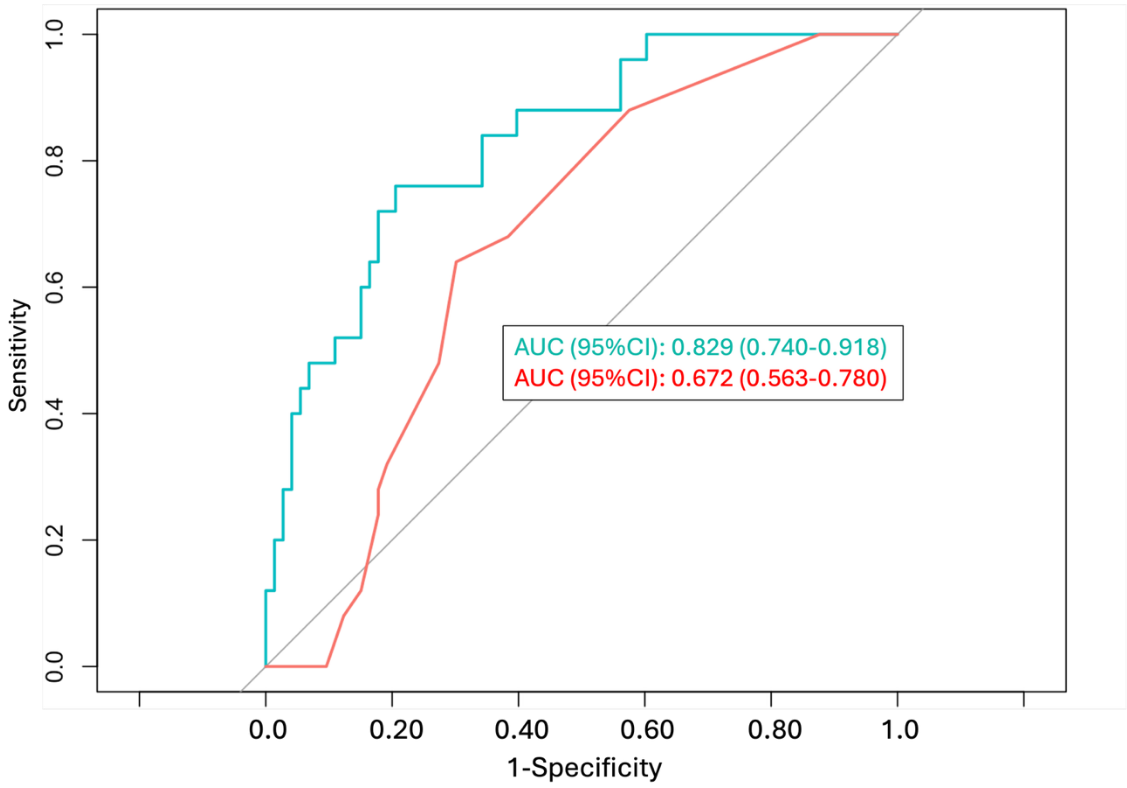

Figure S3. Discrimination for csPCa of the BCN-MRI PM and ERSPC-MRI PM in men suspected of having PCa with PI-RADS 3.

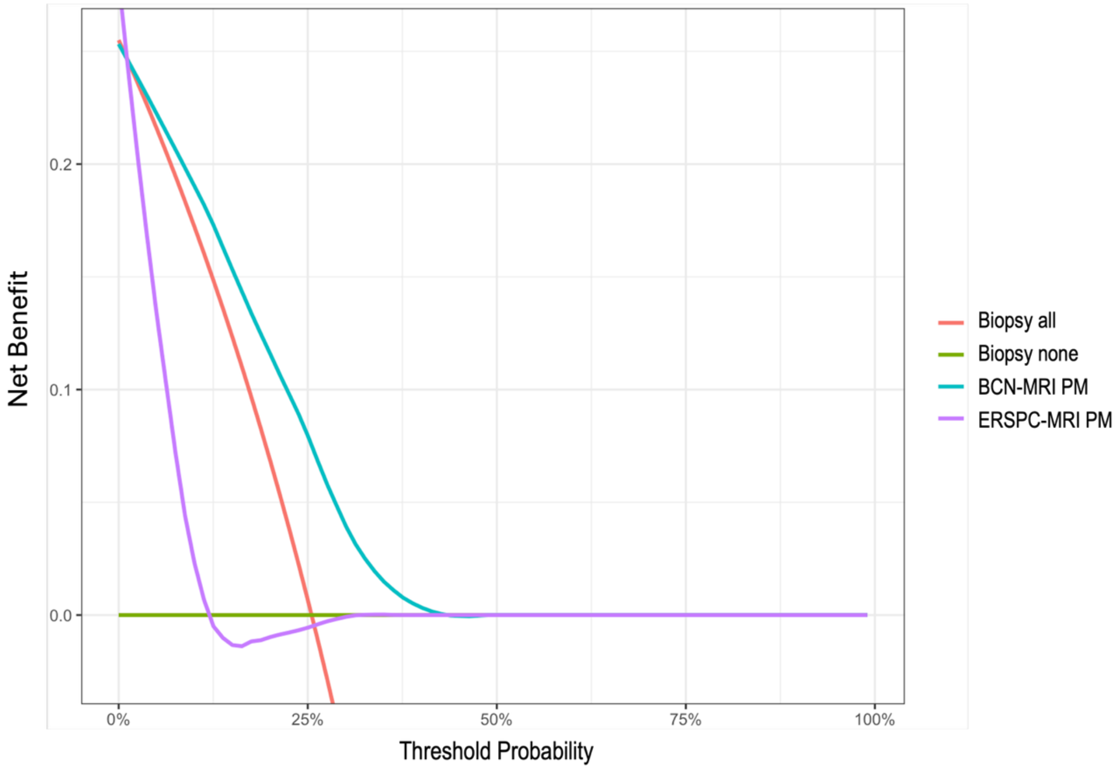

Figure S4. Net benefit over biopsying all men or none of BCN-MRI PM and ERSPC-MRI PM, in men with suspected PCa and PI-RADS 3.

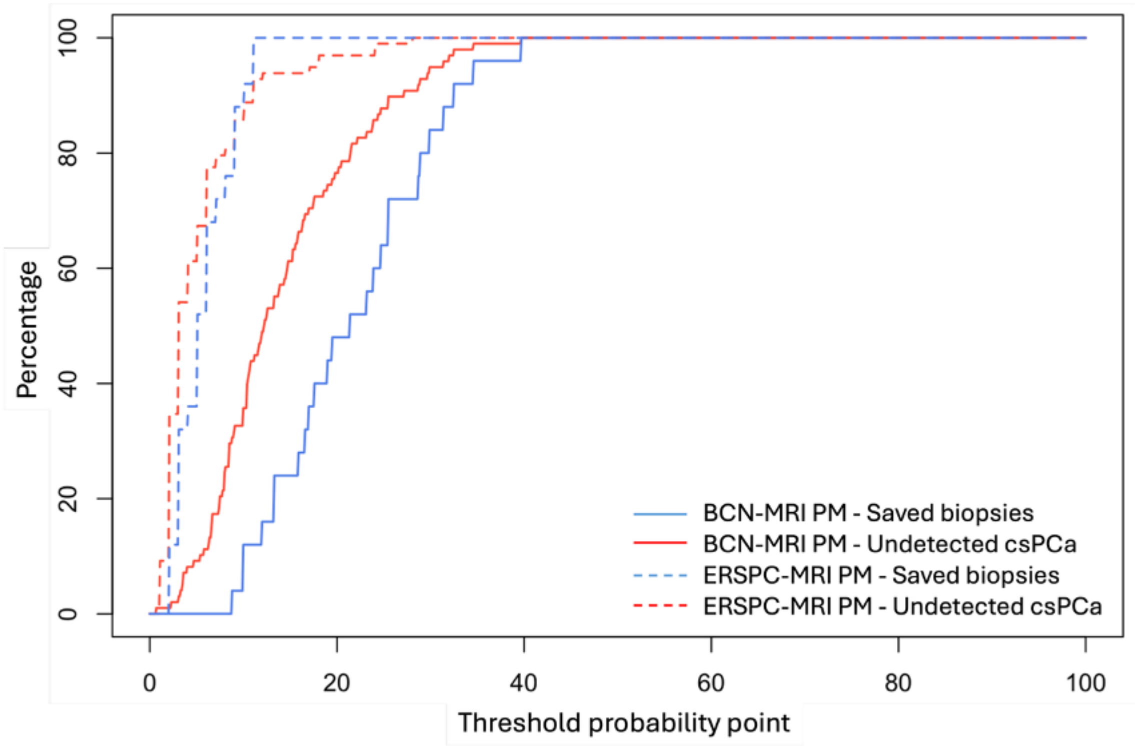

Figure S5. Clinical utility in terms of avoided prostate biopsies and missed csPCa in men with suspected PCa and PI-RADS 3.

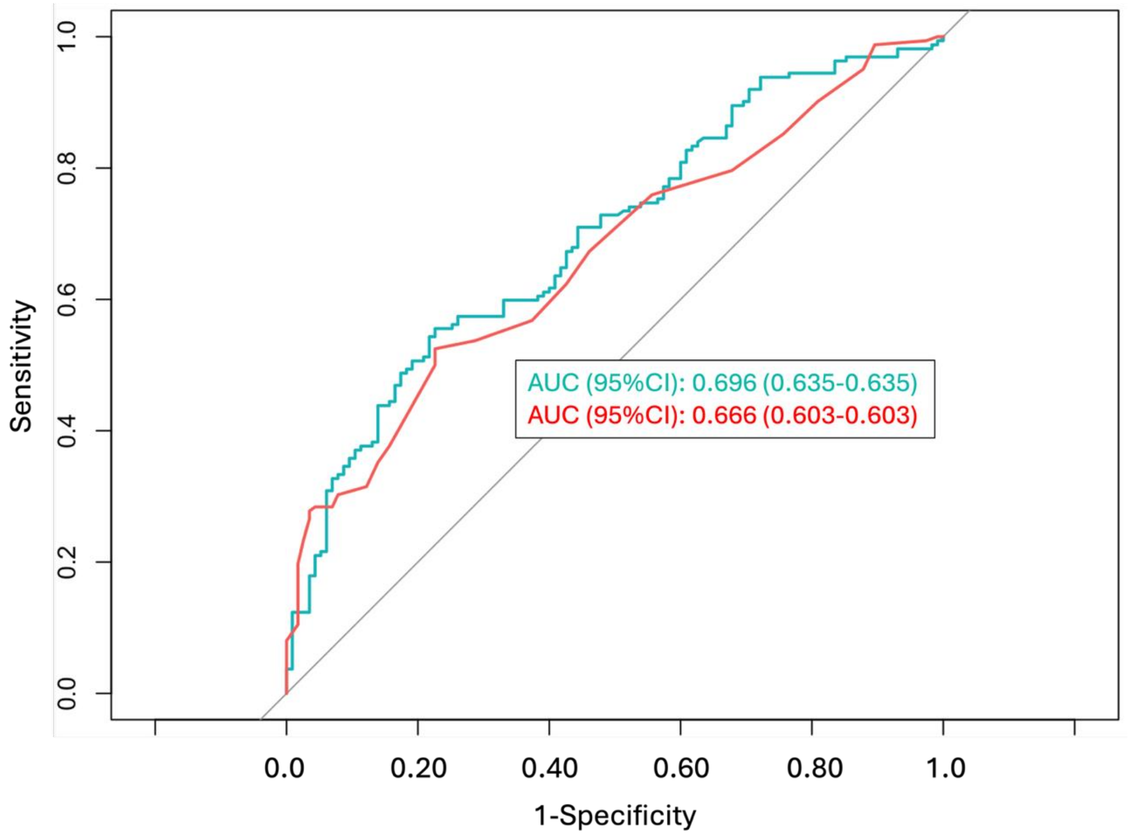

Figure S6. Discrimination for csPCa by the BCN-MRI PM and ERSPC-MRI PM in men suspected of having PCa with PI-RADS 4.

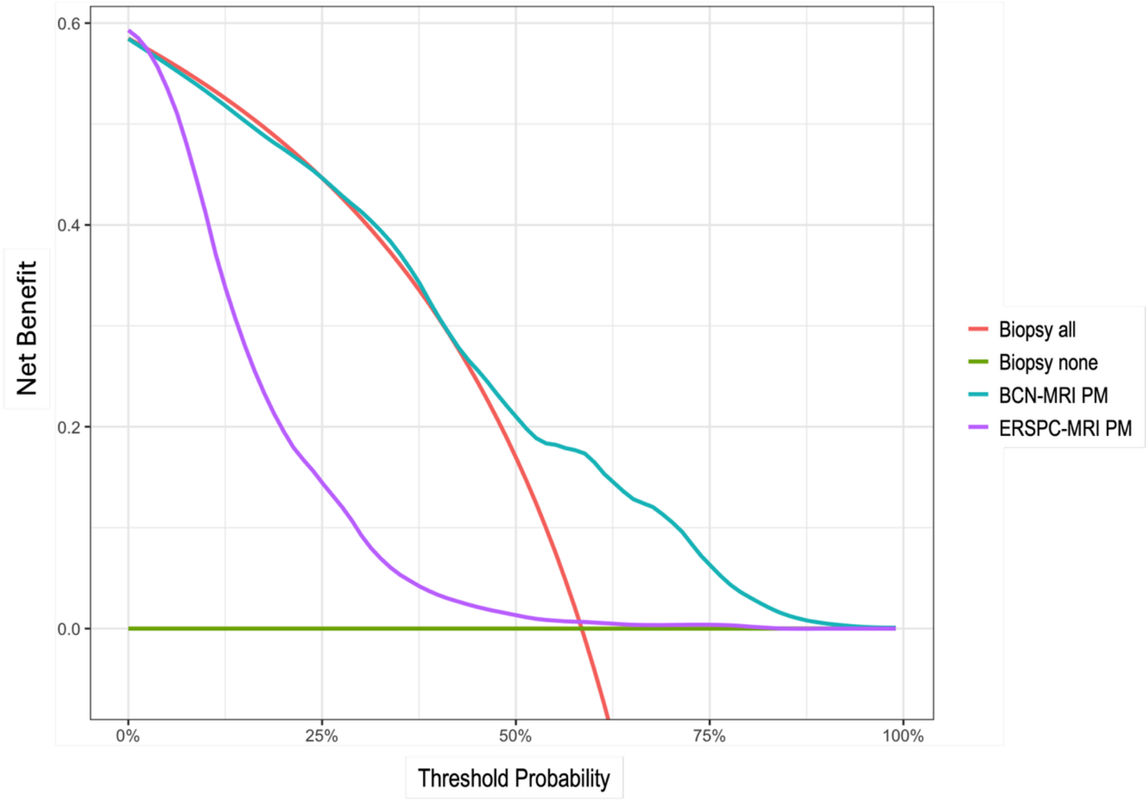

Figure S7. Net benefit over biopsying all men or none of BCN-MRI PM and ERSPC-MRI PM, in men with suspected PCa and PI-RADS 4.

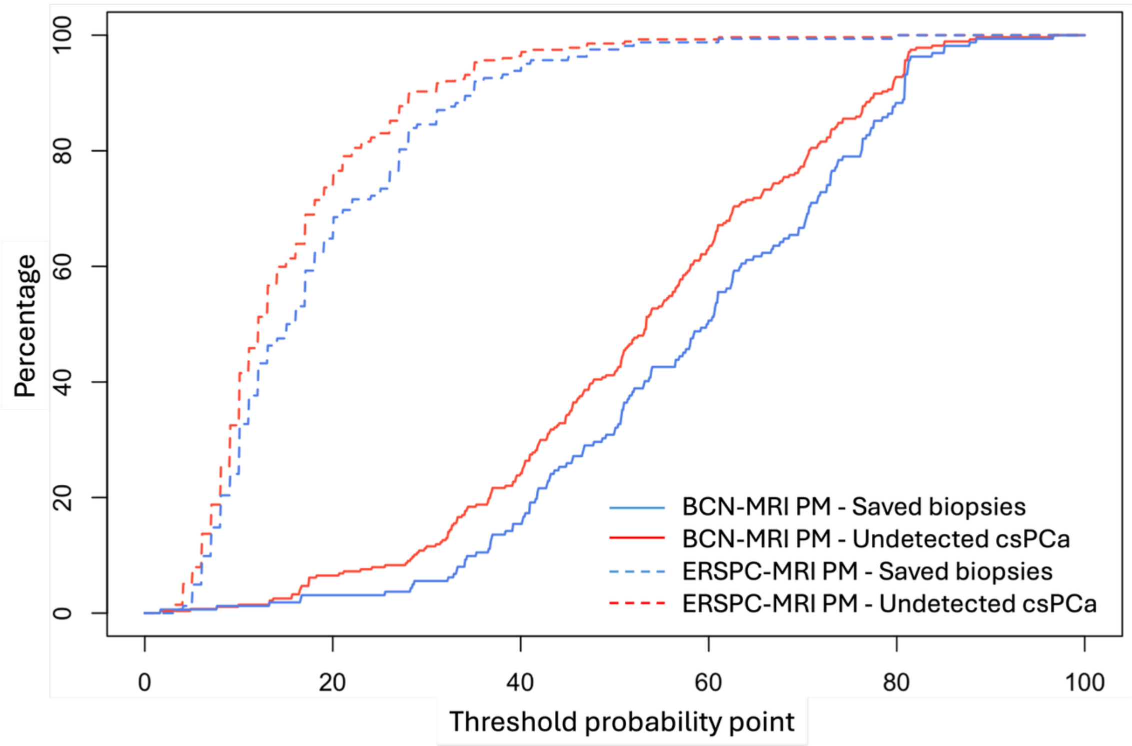

Figure S8. Clinical utility in terms of avoided prostate biopsies and missed csPCa in men with suspected PCa and PI-RADS 4.

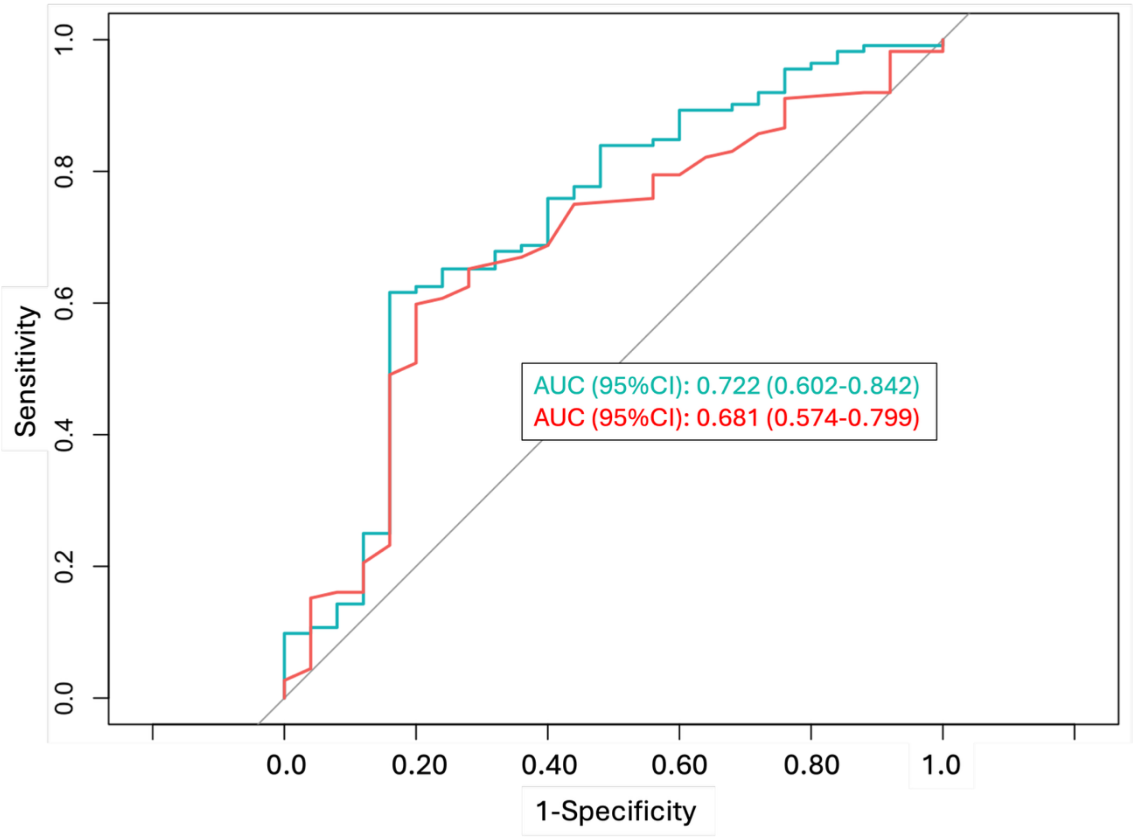

Figure S9. Discrimination for csPCa by the BCN-MRI PM and ERSPC-MRI PM in men suspected of having PCa with PI-RADS 5.

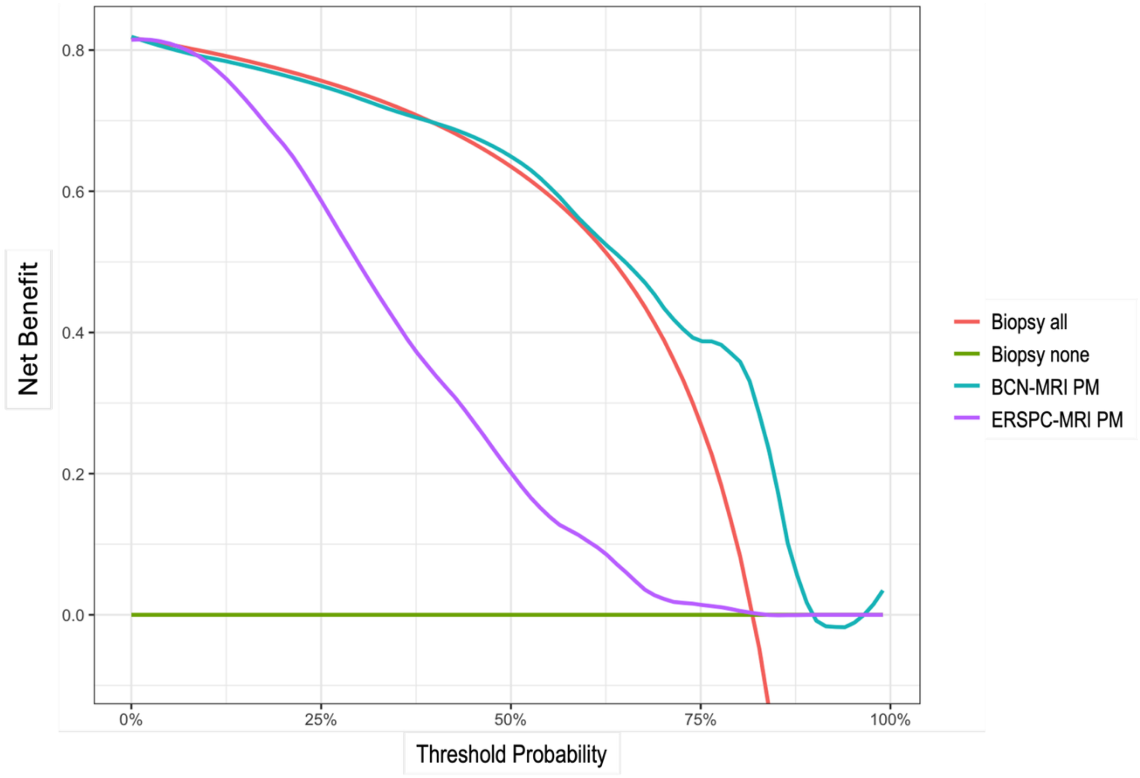

Figure S10. Net benefit over biopsying all men or none of BCN-MRI PM and ERSPC-MRI PM in men with suspected PCa and PI-RADS 5.

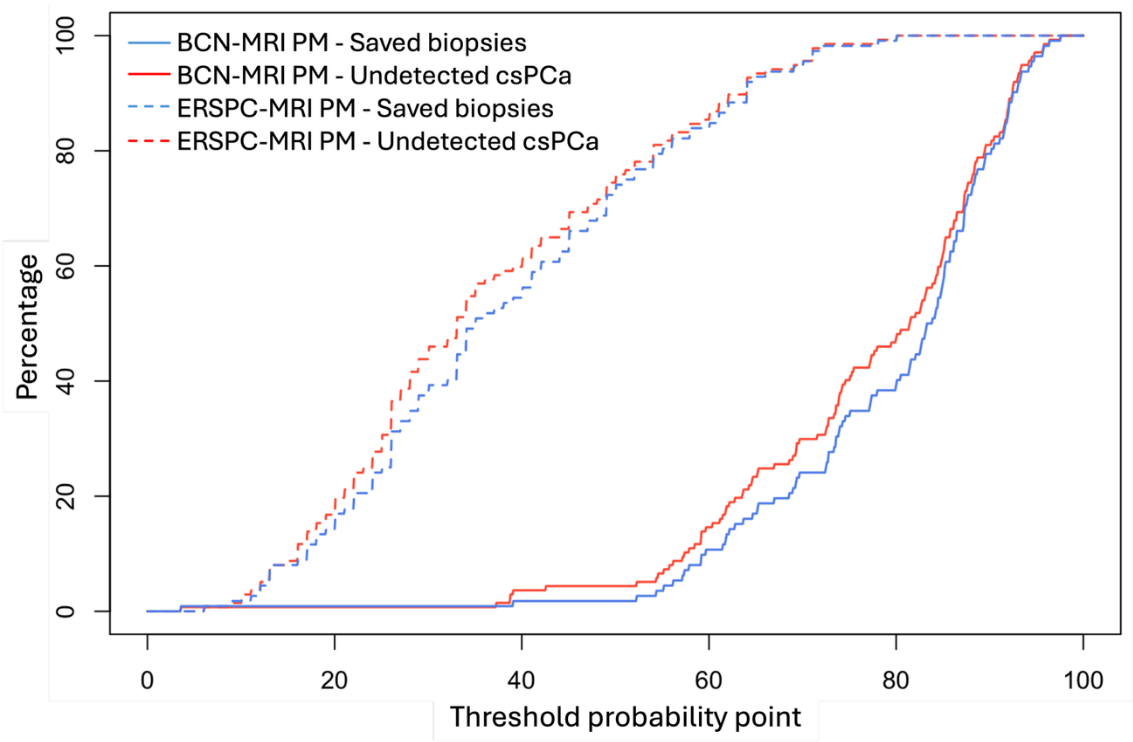

Figure S11. Clinical utility in terms of avoided prostate biopsies and missed csPCa.

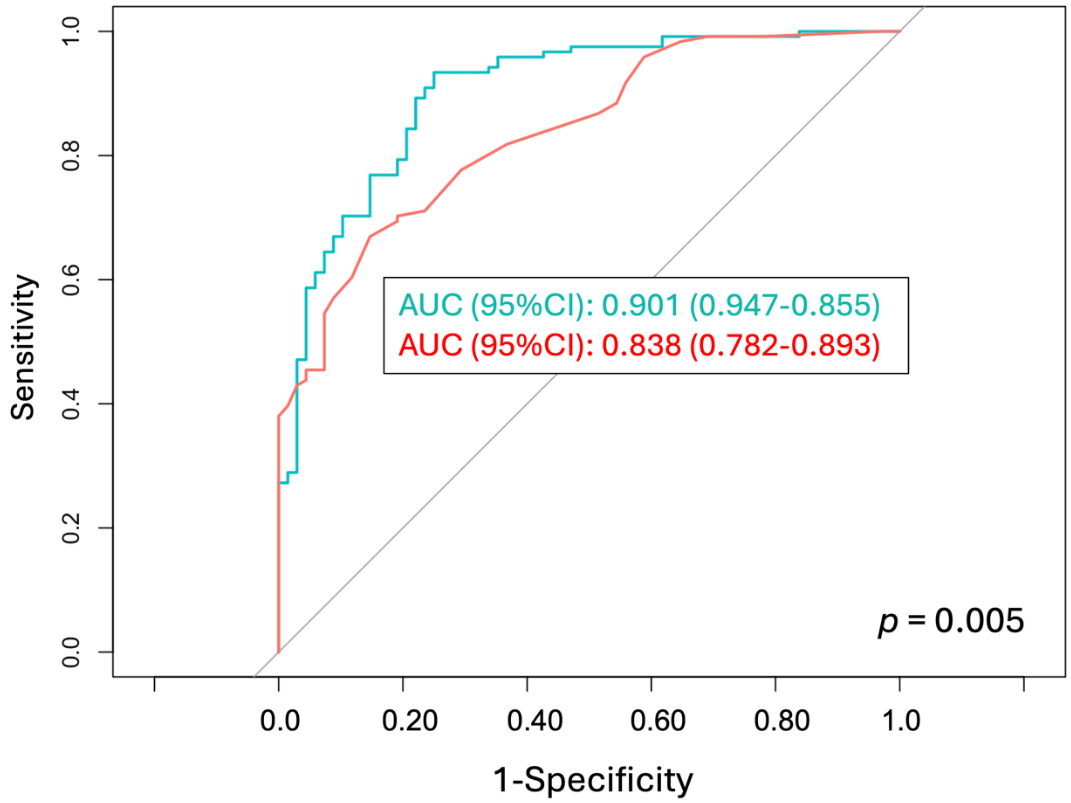

Figure S12. Discrimination for csPCa by the BCN-MRI PM and ERSPC-MRI PM in men suspected of having PCa at Creu Blanca.

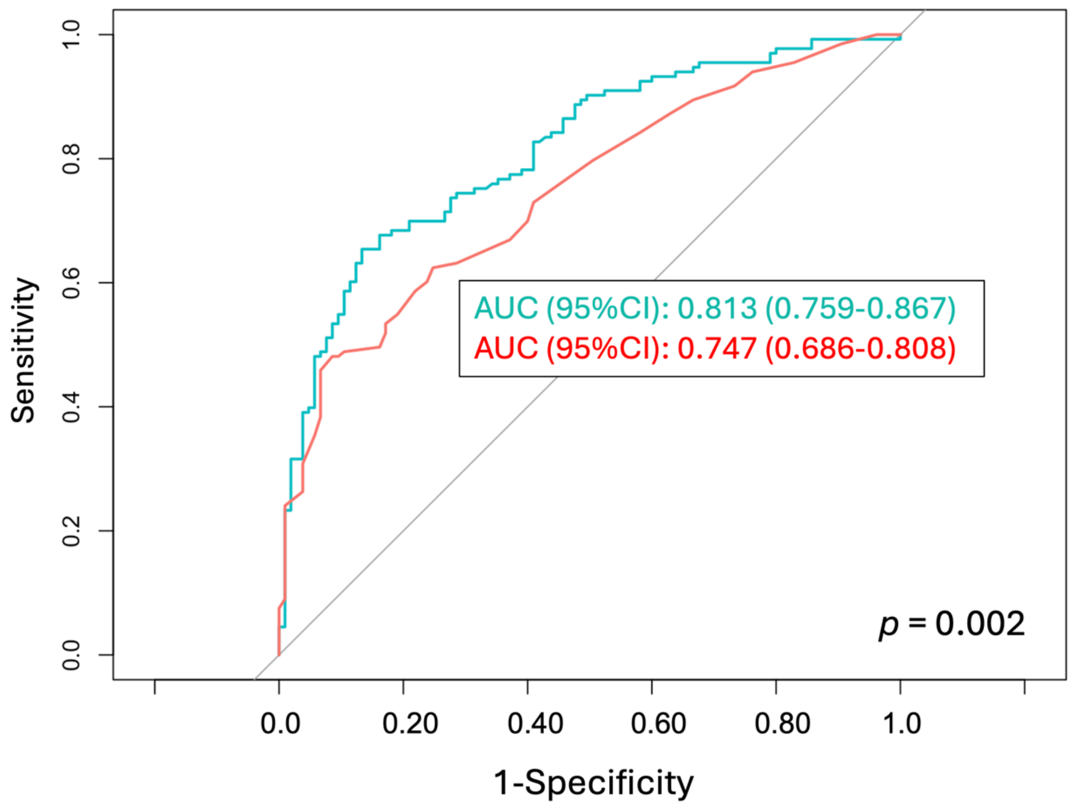

Figure S13. Discrimination for csPCa by the BCN-MRI PM and ERSPC-MRI PM in men suspected of having PCa at Instituto Alexander Fleming.

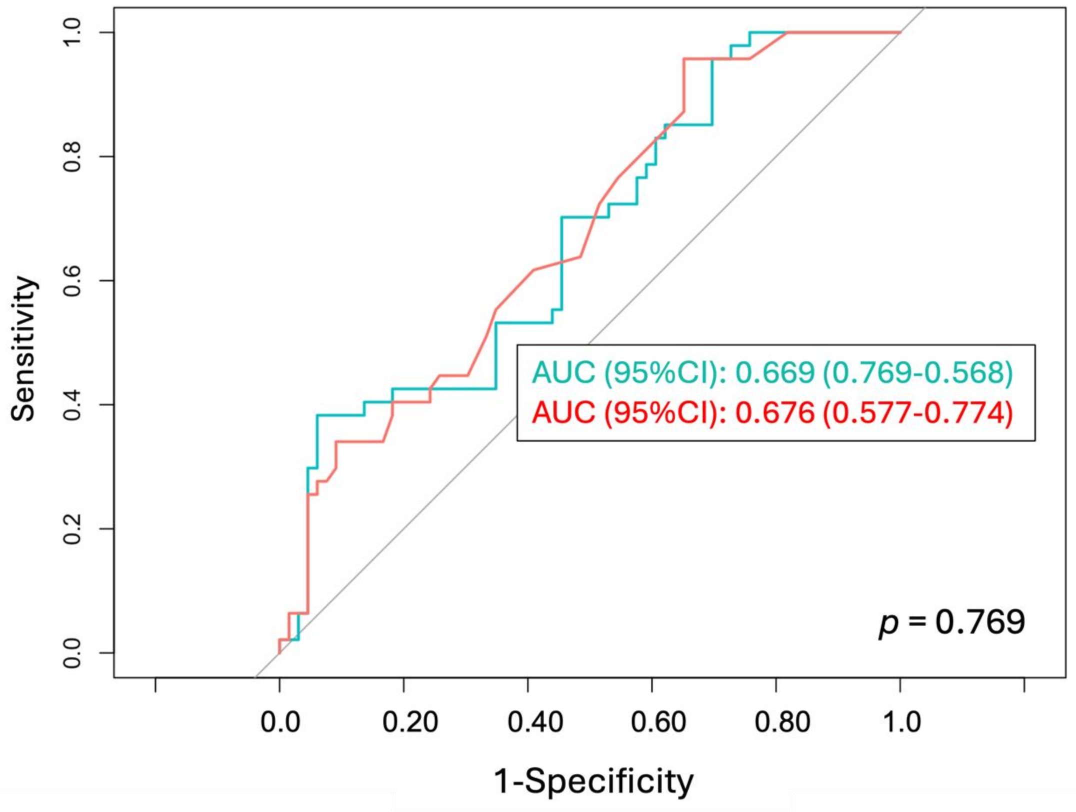

Figure S14. Discrimination for csPCa by the BCN-MRI PM and ERSPC-MRI PM in men suspected of having PCa at Hospital Clínico de la Universidad de Chile.

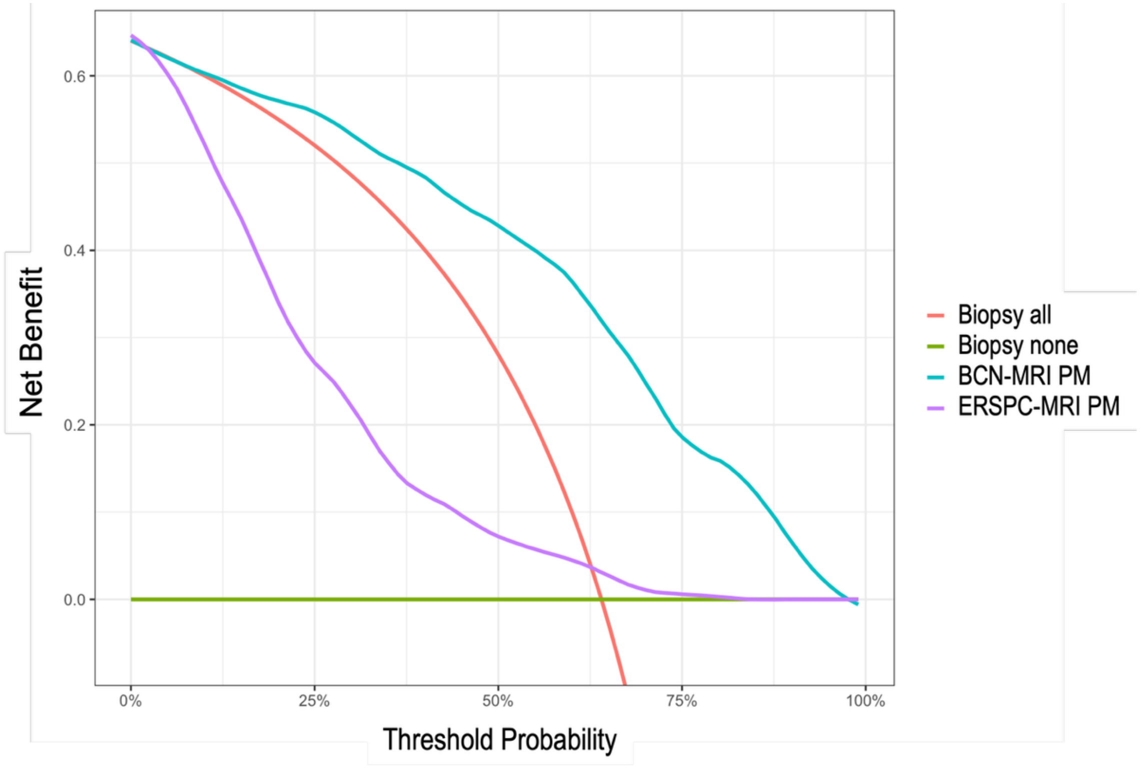

Figure S15. Net benefit of the BCN-MRI PM and ERSPC-MRI PM in men suspected of having PCa at Creu Blanca.

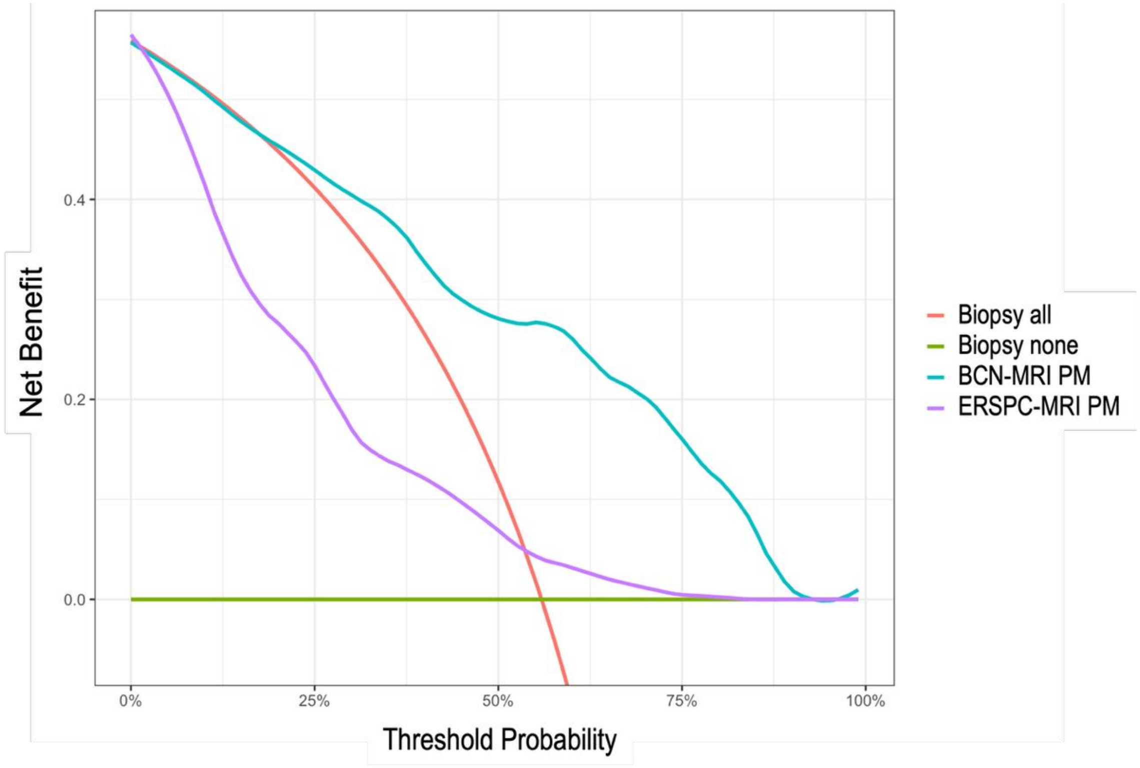

Figure S16. Net benefit of the BCN-MRI PM and ERSPC-MRI PM in men suspected of having PCa at Instituto Alexander Fleming.

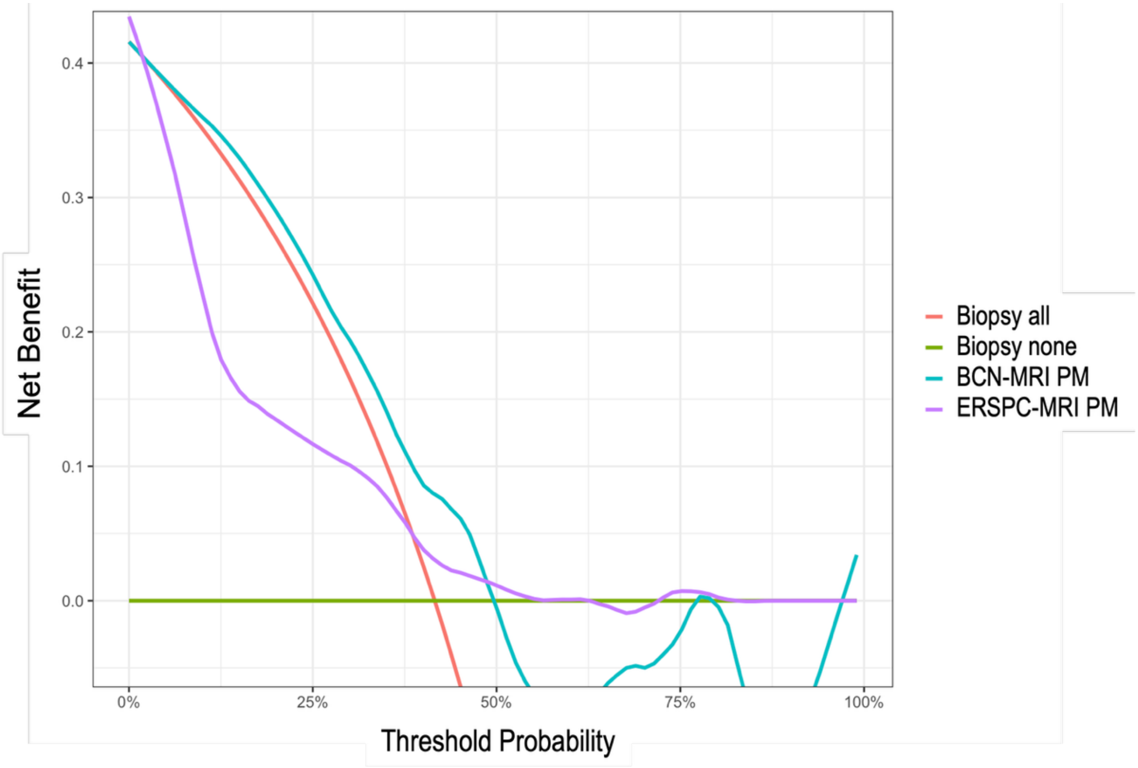

Figure S17. Net benefit of the BCN-MRI PM and ERSPC-MRI PM in men suspected of having PCa at Hospital Clínico de la Universidad de Chile.

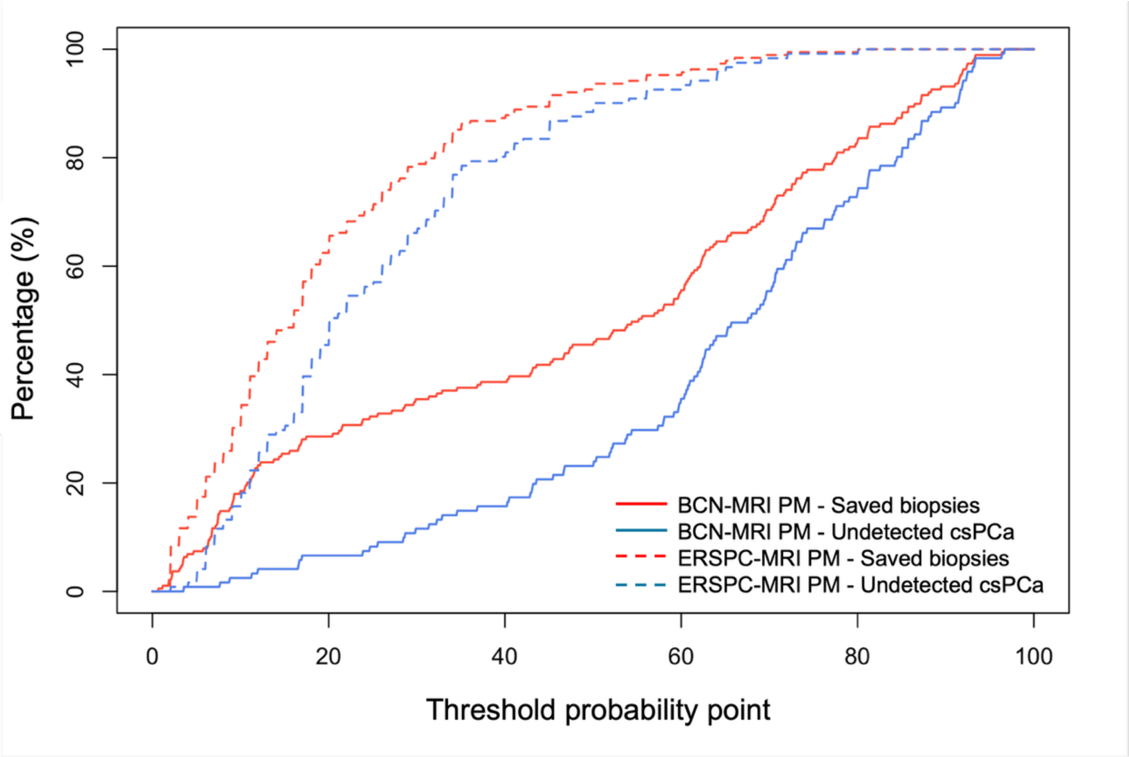

Figure S18. Clinical utility in terms of avoided prostate biopsies and missed csPCa in men from Cruz Blanca.

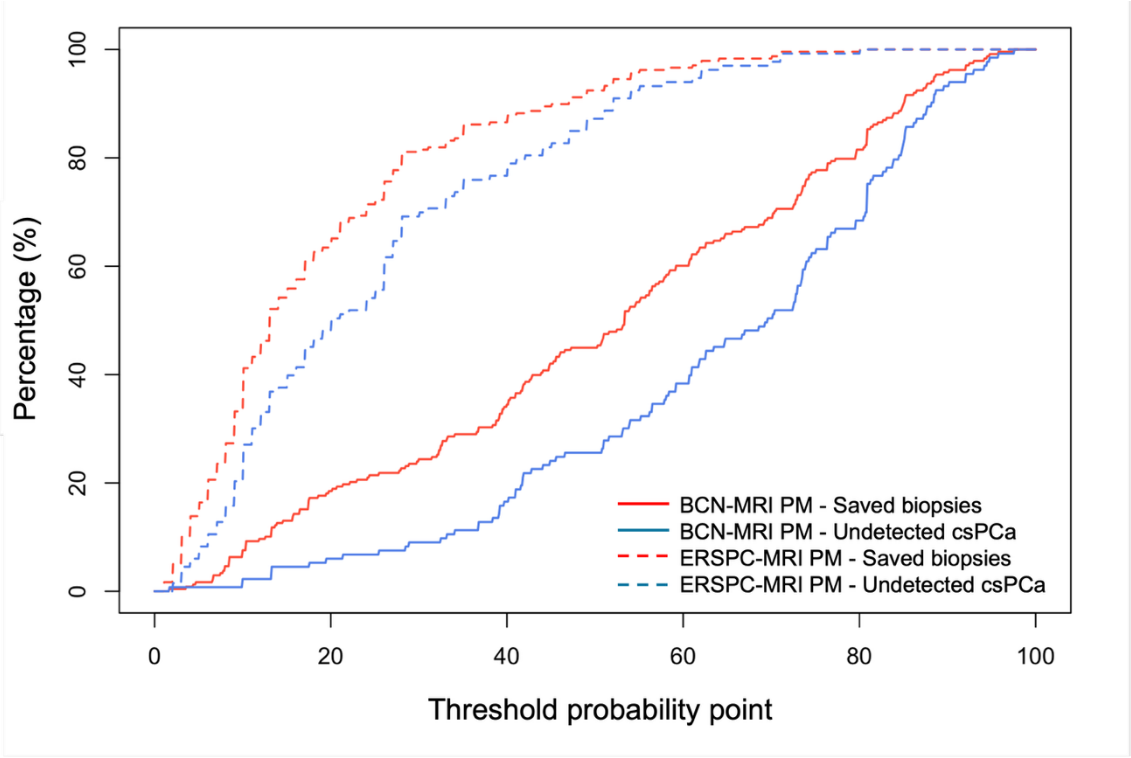

Figure S19. Clinical utility in terms of avoided prostate biopsies and missed csPCa in men from Instituto Alexander Fleming.

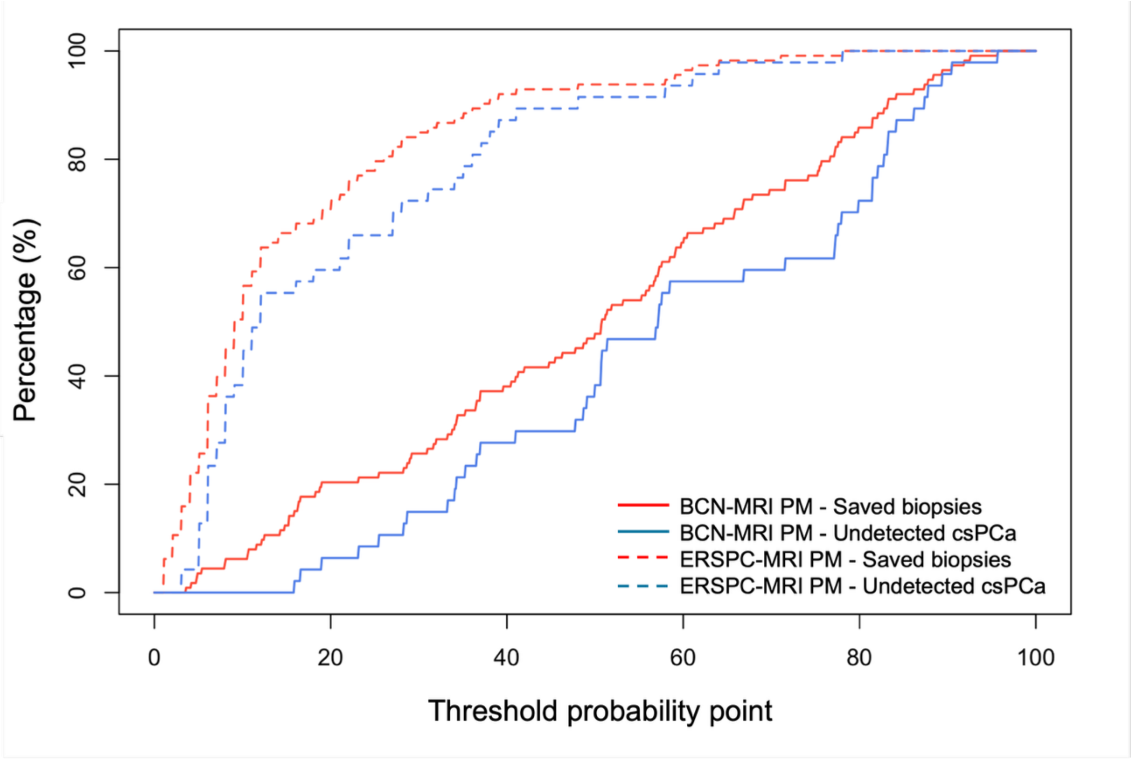

Figure S20. Clinical utility in terms of avoided prostate biopsies and missed csPCa in men from Hospital Clínico de la Universidad de Chile.
